# PEG-RLI: A Long-Acting IL-15 Agonist That Produces Massive Levels of CD8^+^ and CD44^hi^CD8^+^ Cells for Cancer Immunotherapy

**DOI:** 10.3390/pharmaceutics18070863

**Published:** 2026-07-15

**Authors:** Rocio Del Valle Fernandez, Guillermo Hails, John A. Hangasky, Gary W. Ashley, Daniel V. Santi

**Affiliations:** Prolynx Inc., 5858 Horton st, Suite 575, Emeryville, CA 94608, USA; rfernandez@prolynxinc.com (R.D.V.F.); ghails@prolynxinc.com (G.H.); jhangasky@prolynxinc.com (J.A.H.);

**Keywords:** half-life extension, IL-15 agonist, immunotherapy, CD8^+^ T cells, preferential cell expansion

## Abstract

**Background/Objectives**: Interleukin-15 (IL-15) is a promising immunotherapeutic cytokine, but its short half-life limits clinical utility. We developed a stable PEGylated receptor-linker IL-15 agonist (PEG-RLI) to improve pharmacokinetic properties while preserving biological activity and antitumor efficacy. **Methods**: PEG-RLI was synthesized by site-specific N-terminal conjugation of a 40 kDa methoxy polyethylene glycol (MeOPEG) to RLI. The conjugate was characterized for purity and receptor agonism. Pharmacokinetics were evaluated in mice, and pharmacodynamic effects on NK cells and CD8+ T cell subsets were assessed. Antitumor activity was tested in CT26 tumor-bearing mice alone and in combination with anti-PD-1 therapy. **Results**: PEGylation preserved RLI agonistic activity and extended its mouse half-life from approximately 3 h to approximately 15 h. PEG-RLI induced robust expansion of NK cells and CD8^+^ T cells, with preferential enrichment of CD8^+^ T cells and CD44^hi^CD8^+^ T cells. In CT26 solid tumors, PEG-RLI enhanced the antitumor efficacy of anti-PD-1 treatment. **Conclusions**: Stable PEGylation improved the pharmacokinetic profile of RLI while maintaining potent immunostimulatory activity. PEG-RLI preferentially expanded CD8^+^ T cells and showed promising efficacy with immune checkpoint blockade, supporting further development as a differentiated IL-15-based immunotherapeutic.

## 1. Introduction

Interleukin-15 (IL-15) is a cytokine critical for the activation, proliferation, and survival of natural killer (NK) cells and CD8^+^ T cells, making it a key player in immuno-oncology. Endogenous IL-15 is a heterodimeric cytokine that exists in two forms in the body: a tightly bound complex with the IL-15 receptor alpha chain (IL-15Rα) on the surface of an antigen-presenting cell; and the cleaved heterodimeric complex that is circulating in the blood and contributes to homeostasis of the immune system [1,2]. Signal transduction occurs when this IL-15Rα/IL-15 complex binds to the Rβ and Rγ_c_ chains present on the surface of an effector immune cell [3]. Unlike IL-2, IL-15 avoids activation-induced cell death and does not expand immunosuppressive regulatory T cells (T_regs_) [4]. These properties have positioned IL-15 as a promising therapeutic agent for cancer treatment.

Single-chain IL-15 polypeptide has a very short t_1/2_—~1 to 2 h in humans—limiting its effectiveness as a therapeutic agent. A number of larger IL-15 agonists that contain forms of the IL-15/IL-15Rα complex have been made that achieve intermediate half-lives of up to about one day. Applying PEGylation or Fc fusion half-life extension technologies, long-acting IL-15 agonists were developed that prolong half-lives to several days [5,6,7]. Compared with Fc-based approaches, PEGylation avoids introducing an Fc domain that could engage Fc receptors or alter biodistribution while still providing substantial half-life extension. Finally, a depot of a microsphere-IL-15 conjugate tethered by a controlled-release linker shows a very long t_1/2_ of about one week [8]. Notably, in all of these, a cytokine sink may increase the clearance of the IL-15 agonist which may manifest an apparently decreased half-life [9].

Receptor-linker IL-15 (RLI) is a potent IL-15 agonist that fuses human IL-15 to the Sushi domain of IL-15Rα to mimic the natural trans-presentation of IL-15. Unlike IL-15, which requires IL-15Rα for effectiveness, RLI directly binds IL-15Rβγ_c_ with ~100-fold higher affinity than IL-15 and enhances immune effector cells—both NK and CD8^+^ T cells [10,11]. However, as with most other IL-15 agonists, the relatively short half-life of RLI of ~3 h restricts its utility. We recently prepared a slow-release long-acting microsphere depot of RLI, but necrotic injection site reactions prohibited its use [12]. We hypothesized that the long subcutaneous (SC) residence time and continuous local release of RLI from the insoluble depot induced a strong local immune activation. Recognizing that a soluble RLI conjugate would not have as long an SC residence time as the insoluble depot, we posited that a releasable PEGylated RLI might be a safer source of systemic RLI. We prepared a releasable PEG-RLI by attaching a cleavable linker to the N-terminus of RLI that would slowly release free RLI from PEG; we likewise prepared a non-releasable, stable PEG-RLI conjugate as a control. Surprisingly, we found that the stable PEG-RLI control caused very high expansion of immune cells.

In the present work, we describe the synthesis and characterization of stable PEG-RLI, a stable conjugate of 40 kDa MeOPEG with RLI. First, we describe the chemistry used for site-specific N-terminal PEGylation of the IL-15 agonist, RLI, and show that it maintains full agonistic activity to the IL-15Rβγ_c_. Next, we describe the pharmacokinetics (PK) of PEG-RLI in mice and show that PEGylation extends RLI’s t_1/2_ from ~3 to ~15 h in mice. Notably, PEG-RLI displays pharmacodynamic (PD) properties in which it preferentially expands CD8^+^ T cells over NK cells, resulting in very high frequencies of CD8^+^ and CD44^hi^ CD8^+^ T cells. Finally, we show that PEG-RLI in combination with anti-PD-1 has antitumor efficacy in CT26 solid tumors.

## 2. Materials and Methods

Complete, detailed descriptions and procedures used for the synthesis and characterization of PEG-RLI, PK and PD studies are provided in the Supplementary Information.

### 2.1. Preparation of PEG–RLI Conjugates

RLI was site-selectively modified via reductive alkylation with N_3_-PEG_4_-CHO in citrate buffer at pH 6.0 using NaCNBH_3_ as the reducing agent, yielding a mixture of mono- and di-alkylated species, as confirmed by SDS–PAGE. Reaction conditions were optimized, varying the N_3_-PEG_4_-CHO: RLI molar ratio. The azido RLI intermediates were subsequently reacted with MeOPEG-BCN under mild click conditions to form PEG–RLI conjugates. Excess PEG–BCN was quenched and removed by azido-agarose bead capture and filtration. Alternatively, MeO-PEG-CHO was used for alkylation of RLI to directly yield PEG-RLI. PEG–RLI was purified by anion-exchange chromatography, concentrated, and buffer-exchanged into PBS. Product identity and purity (>95%) were confirmed by SDS–PAGE, with free RLI below the limit of detection. Scale-up reactions (up to 40 mg RLI) were performed under analogous conditions, affording purified PEG–RLI in a 15–25% yield.

### 2.2. Cell-Based Assay

The biological activity of PEG-RLI was evaluated using a U2OS cell-based assay kit for IL-2/IL-15Rβγ_c_ binding following the manufacturer’s instructions (DiscoverX, Fremont, CA, USA, Part #93-0998E3CP5).

### 2.3. Animal Welfare

All animal studies were conducted under protocols approved by the Institutional Animal Care and Use Committees of MuriGenics (Vallejo, CA, USA) and Explora Biolabs (San Francisco, CA, USA), in accordance with institutional and federal ethical regulations.

### 2.4. In Vivo Pharmacokinetic and Pharmacodynamic Studies

For PK studies, PEG-RLI was administered SC or IV to male C57BL/6J mice. Alternating groups of animals were bled, and blood samples were collected in EDTA collection tubes containing HALT protease inhibitors. Plasma samples were analyzed by the Simple Plex Human IL-15 Cartridge (Biotechne, Minneapolis, MN, USA, Cat #SPCKB-PS-000500). A standard curve with PEG-RLI was performed as a concentration reference. For PD studies, PEG-RLI conjugates were administered SC to male C57BL/6J mice. Whole blood and/or spleens were collected at different times depending on the experiment (dose titration or longitudinal PD studies). PBMCs and splenocytes were immunophenotyped by flow cytometry. Body weights were determined twice a week.

### 2.5. Flow Cytometry

For PD studies in mice, whole blood (EDTA) was labeled with a fixable viability dye, blocked with anti-CD16/32, and stained with optimized antibody panels for PBMC markers (Table S1). RBCs were lysed and cells fixed/permeabilized for intracellular staining. Splenocytes were prepared as single-cell suspensions, RBC-lysed, Fc-blocked, and stained similarly, with fixation and permeabilization using the Foxp3 Transcription Factor Staining Buffer Set (Invitrogen, Carlsbad, CA, USA). Samples were acquired on an Attune NxT flow cytometer (BD Biosciences, San Jose, CA, USA) and analyzed using FlowJo (TreeStar, Ashland, OR, USA). Cell numbers were determined by volumetric counting. The gating strategy was as follows: Lymphocytes were first gated based on forward and side scatter (FSC-A vs. SSC-A), followed by singlet discrimination using FSC-A vs FSC-H. Live cells were identified using a fixable viability dye. Live CD19^−^ cells were gated to exclude B cells. T cells were defined as CD3^+^ cells and subdivided into CD4^+^ and CD8^+^ subsets, with CD44 expression assessed within the CD8^+^ compartment. NK cells were identified as NK1.1^+^ cells within the CD3^−^ population (Figure S12 [12]); FMO controls were used to define positive populations. CD8^+^ T cells were identified as CD3^+^CD4^−^CD8^+^, CD44^hi^CD8^+^ T cells as CD3^+^CD4^−^CD8^+^CD44^hi^, NK cells as CD3^−^NK1.1^+^, and proliferating cells as Ki67^+^.

### 2.6. CT26 Syngeneic Model

CT26 tumors were established in female BALB/c mice by SC inoculation of 1 × 10^5^ cells. Mice with established tumors (~50 mm^3^) were randomized into treatment groups (n = 6–7/group): untreated control (PBS), anti–PD-1 (200 µg, intraperitoneal, BioXcell clone RMP1-14, on D0, D5, D7, D13, D17), RLI or PEG–RLI alone (0.5 mg/kg, s.c., D4), and RLI or PEG-RLI plus anti-PD-1 combinations. Tumor volumes were measured twice weekly, and survival was monitored for 60 days. Kaplan–Meier mouse survival plots were generated based on the mouse survival, monitored based on humane end point criteria. The tumor volume was measured by calipers and calculated by the following equation: V = 1/2(long dimension)(short dimension).

### 2.7. Statistics

The statistical tests used are indicated in the corresponding figure legends. One-way analysis of variance (ANOVA) followed by Tukey’s multiple comparisons test was used when a single independent variable was analyzed. For longitudinal studies, two-way ANOVA or mixed-effects models were applied, followed by Tukey’s or Dunnett’s post hoc multiple comparisons tests, as appropriate. Tumor volumes are presented as median ± median absolute deviation (MAD) and were analyzed using two-way ANOVA with Tukey’s multiple comparisons test. Kaplan–Meier survival curves were compared using the log-rank (Mantel–Cox) test. All statistical analyses were performed using GraphPad Prism (v10.6.1). A *p* value ≤ 0.05 was considered statistically significant.

## 3. Results

**Scheme 1:**
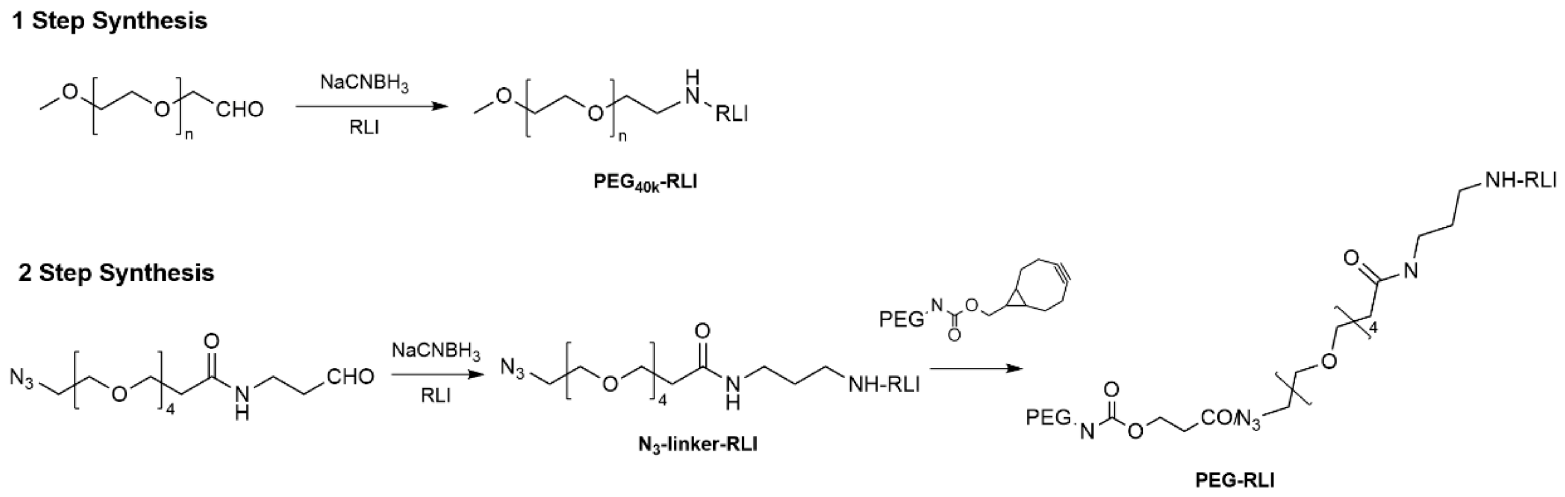
Synthesis of PEG-RLI.

Site-specific PEGylation of RLI. PEGylated conjugates of RLI were prepared by reductive alkylation of the N-terminus of RLI with an appropriate PEG-aldehyde and NaCNBH_4_ (Scheme 1; Figure S1) or by the two-step procedure described below. One-step reactions were performed by reductive alkylation of RLI with 20 kDa or 40 kDa linear or 40 kDa branched MeOPEG_n_-CHO. SDS-PAGE gel shift analysis (Table 1) showed that reactions using five equivalents of MeOPEG-CHO resulted in 33- to 67% mono-PEGylated RLIs, representing ~82–90% of the total N^α^-PEGylated protein. AEX was used to purify mono-PEGylated RLIs to greater than 95% purity. In the alternative two-step preparation, RLI was first alkylated with N_3_-PEG_4_-CHO and then the mixture conjugated to MeOPEG_40kDa_-BCN through SPAAC. SDS PAGE gel shift analysis [13] showed the highest yield of the mono-PEGylated protein was obtained using one equivalent of N_3_-PEG_4_-CHO; ~34% of the total RLI was mono-acylated, representing ~81% of the total acylated RLI. When scaled to 5 mg RLI, after purifying by AEX chromatography, the mono-PEGylated RLI was obtained in a 24% overall yield and was ≥95% pure. The PEG-RLI used in the ensuing experiments contained a linear 40 kDa MeOPEG (i.e., MeOPEG_40kDa_-RLI) and was prepared by both one- and two-step reactions; unless otherwise specified, the studies described used material prepared by the latter approach.

**Figure 1:**
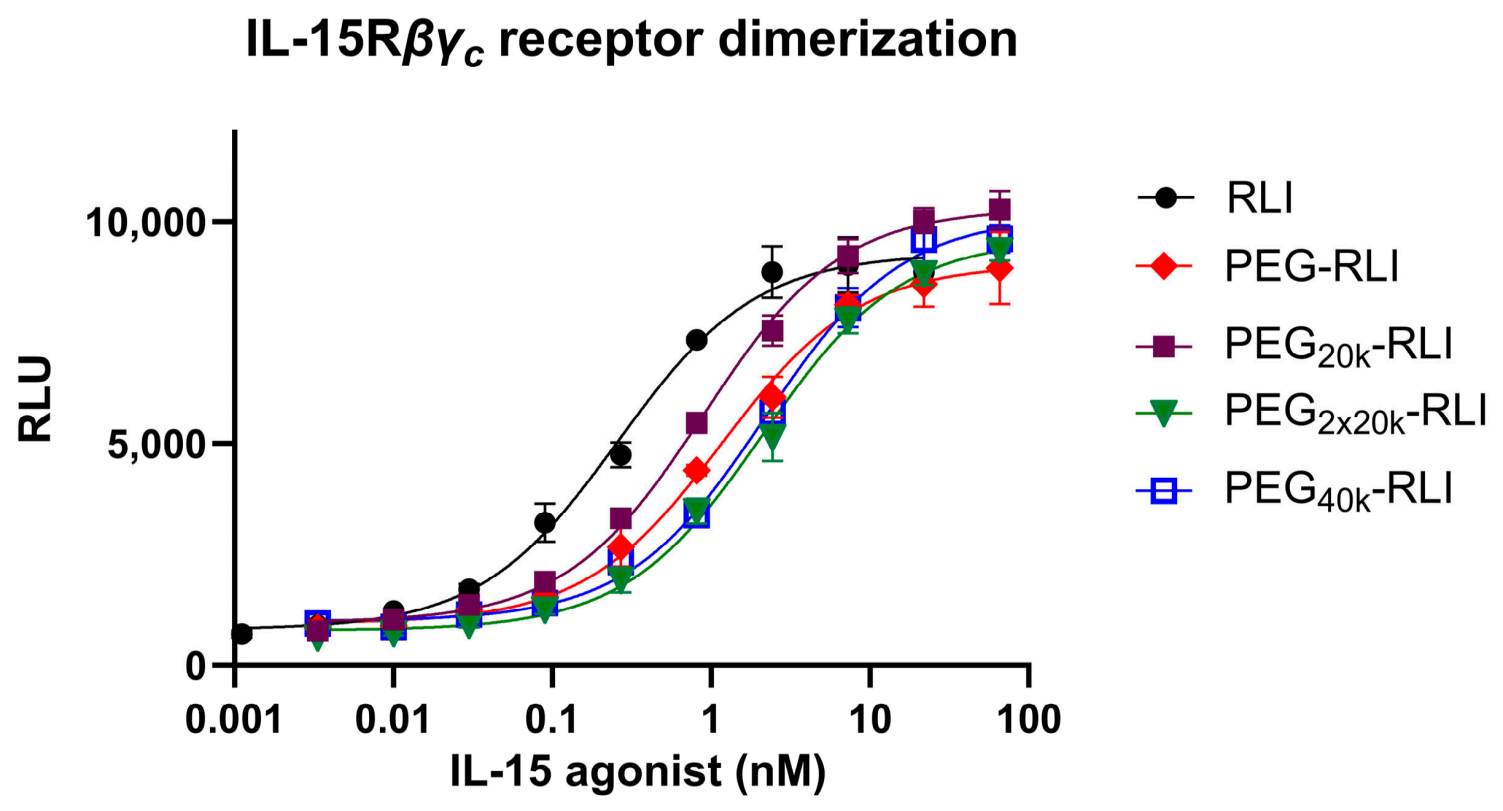
Bioactivity of RLI and N-PEG-RLIs. Cell-based assay measuring the dimerization of IL-2/15Rβγ_c_ when incubated with free RLI (⬤, EC_50_ = 0.26 nM), PEG-RLI (◆, EC_50_ = 1.2 nM), PEG_20kDa_-RLI (■, EC_50_ = 0.9 nM) PEG_40kDa_-RLI (□, EC_50_ = 2.2 nM) or PEG_2x20kDa_-RLI (▼, EC_50_ = 2.1 nM). PEG-RLI (◆) was prepared by reductive alkylation with N_3_-PEG_4_-CHO followed by SPAAC to PEG_40k_-BCN. All other conjugates were prepared by reductive alkylation using MeOPEG-CHO. Data were fitted to a three-parameter logistic model. Points represent the mean ± SD.

In vitro characterization of PEG-RLI. The potency and stability of conjugates of PEG and RLI were assessed using an engineered U2OS dimerization cell line expressing the IL-2/IL-15Rβγ_c_ receptor. As shown in Figure 1, conjugates of PEG and RLI and unmodified RLI exhibited full agonist activity at saturating concentrations. The EC_50_ values for various PEGylated RLIs ranged from 1.2 to 2.2 nM, ~5 to 8 times higher than those for unmodified RLI. PEG-RLI retained 100% of its initial potency after incubation in PBS at 37 °C for ten days, with no detectable degradation products observed via SDS-PAGE analysis (Figure S5).

**Figure 2:**
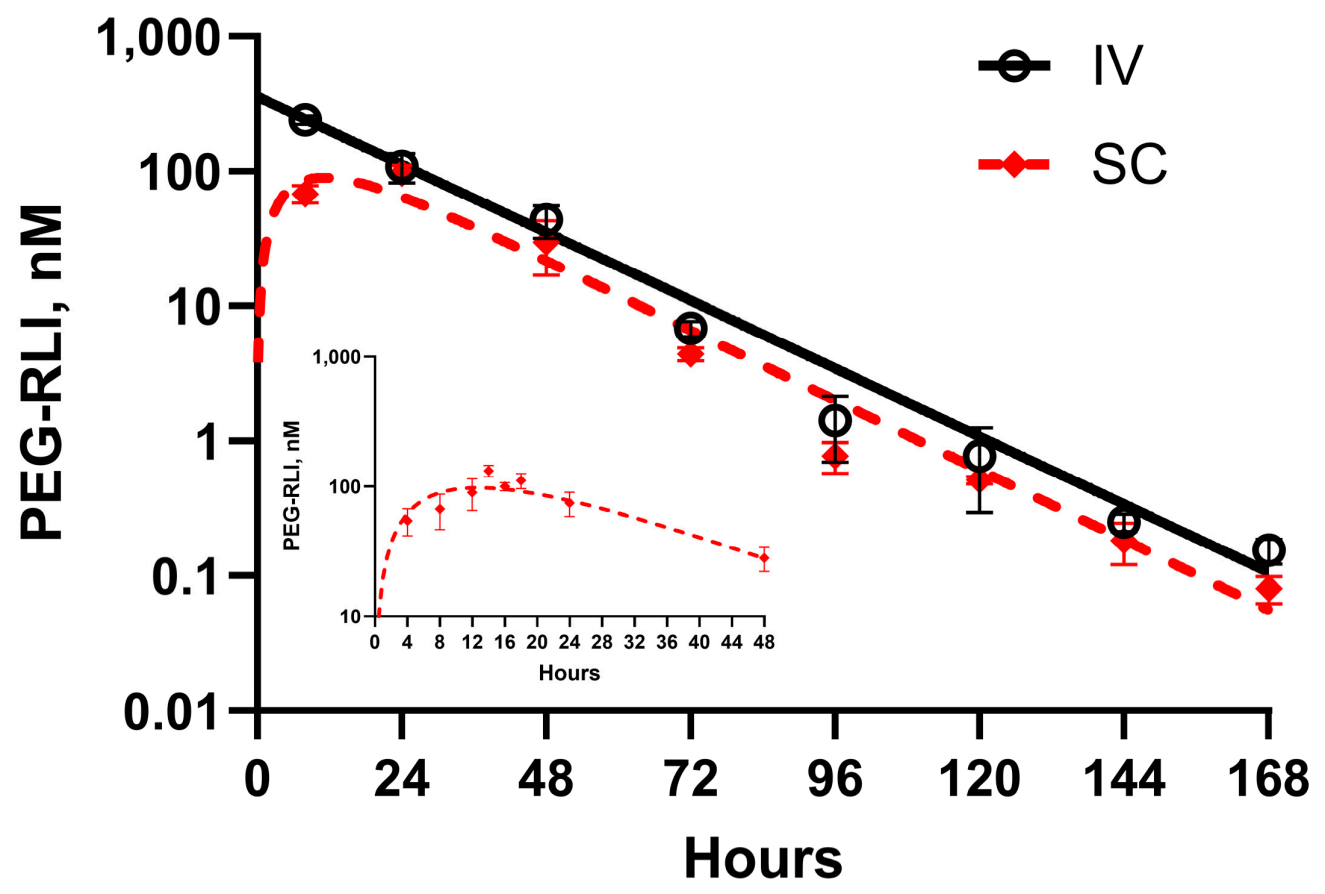
C vs. t plot of PEG-RLI. C57BL/6J mice were administered PEG-RLI (10 µg PEG-RLI, 0.43 nmol) IV(◯) or SC (◆). PEG-RLI plasma concentrations were determined by ELISA. The inset is a separate experiment showing the first 48 h; data was fitted to a biexponential decay model with the following parameters: k_1_ = 0.051 h^−1^ (t_1/2_ = 14 h); k_2_ = 0.114 h^−1^ (absorption t_1/2_ = 6 h); t_max_ = 14 h; C_max_ = 133 nM. Data points represent mean ± SD (n = 6/group).

PK of PEG-RLI in mice. Figure 2 and Table 2 show the PK parameters of PEG-RLI given by intravenous (IV) and SC injections. Due to renal retardation of 40 kDa PEG conjugates, we surmised PEG-RLI would exhibit an extended terminal t_1/2_ and increased exposure compared to unmodified RLI. Following IV administration of 10 µg PEG-RLI to mice, a C_Max_ of 240 nM was observed with a t_max_ of 8 h and an elimination t_1/2_ = 15 h. SC administration resulted in a lower C_Max_ (97 nM) and longer t_max_ (14 h) but maintained the same elimination t_1/2_ (15 h). The bioavailability following SC administration was 46% based on area under the curve (AUC) values. The lower SC bioavailability likely reflects slow diffusion of PEG-RLI from the SC injection site into systemic circulation [14]. Overall, PEGylation increased the t_1/2_ by ~5-fold compared to unmodified RLI (t_1/2_ = 15 vs. 3 h) [15]. A 10 μg injection IV or SC gives normalized C_max_ values of 24 nM/µg (C_max_/dose) or 9.7 nM/µg, respectively. So, a 10 μg SC dose should retain a concentration of >1 nM PEG-RLI for six half-lives or ~4 d, and an IV injection would remain >1 nM for eight half-lives or ~5 d.

Table 2 also compares the PK parameters of PEG-RLI to those reported for NKTR 255, long-acting depots of MS~RLI and MS~IL-15, and the free RLI and IL-15 proteins. The t_1/2_ and clearance values for PEG-RLI are almost identical to those reported for NKTR-255. In contrast, PEG-IL-15 and RLI have a higher C_max_ and AUC than the other agonists. The AUC of 10 µg PEG-RLI is 3600 nM∗h, compared to 6- to 7 nM∗h for RLI from 10 ug MS-RLI or MS~IL-15. This is not surprising since the clearance of PEG_40kDa_-agonists is much reduced, which in effect raises the concentration of the agonist compared to the same doses of the higher clearance and smaller proteins [16]. Interestingly, the AUC values for the PEGylated IL-15 agonists are ~100-fold higher than the other agonists shown.

Maximum tolerated dose (MTD). In naïve C57BL/6J mice, subcutaneous administration of PEG-RLI at doses ranging from 4 to 20 µg was well tolerated, resulting in <5% body weight loss (BWL) immediately following treatment and subsequent steady weight gain over an 18-day observation period (Figure S8). In contrast, mice receiving 40 µg PEG-RLI exhibited approximately 20% BWL by day 5, accompanied by a marked reduction in immune cell numbers (Figure S7) and clinical signs of lethargy, indicating that the maximum tolerated dose had been exceeded. No injection-site necrosis was observed at any dose tested (4–40 µg). Similar findings were obtained with PEG-RLI generated by one-step synthesis (PEG_40kDa_-RLI), where 20 and 40 µg doses induced approximately 10% BWL by day 5, while the 10 µg dose produced less than 10% weight reduction (Figure S10A). Mice treated with 10 µg recovered to baseline body weight during the observation period, whereas those receiving 40 µg remained approximately 10% below baseline through day 14 (Figure S10A). Consistent with these observations, plasma cytokine analysis on day 5 demonstrated transient, dose-dependent increases in TNF-α, IL-6, IFN-γ, and IL-10 at the 20 and 40 µg dose levels (Figure S10B–G), indicative of systemic inflammatory responses. Collectively, these data establish that PEG-RLI doses of 10 µg or less are well tolerated and do not elicit measurable cytokine-associated toxicities under the conditions evaluated.

**Figure 3:**
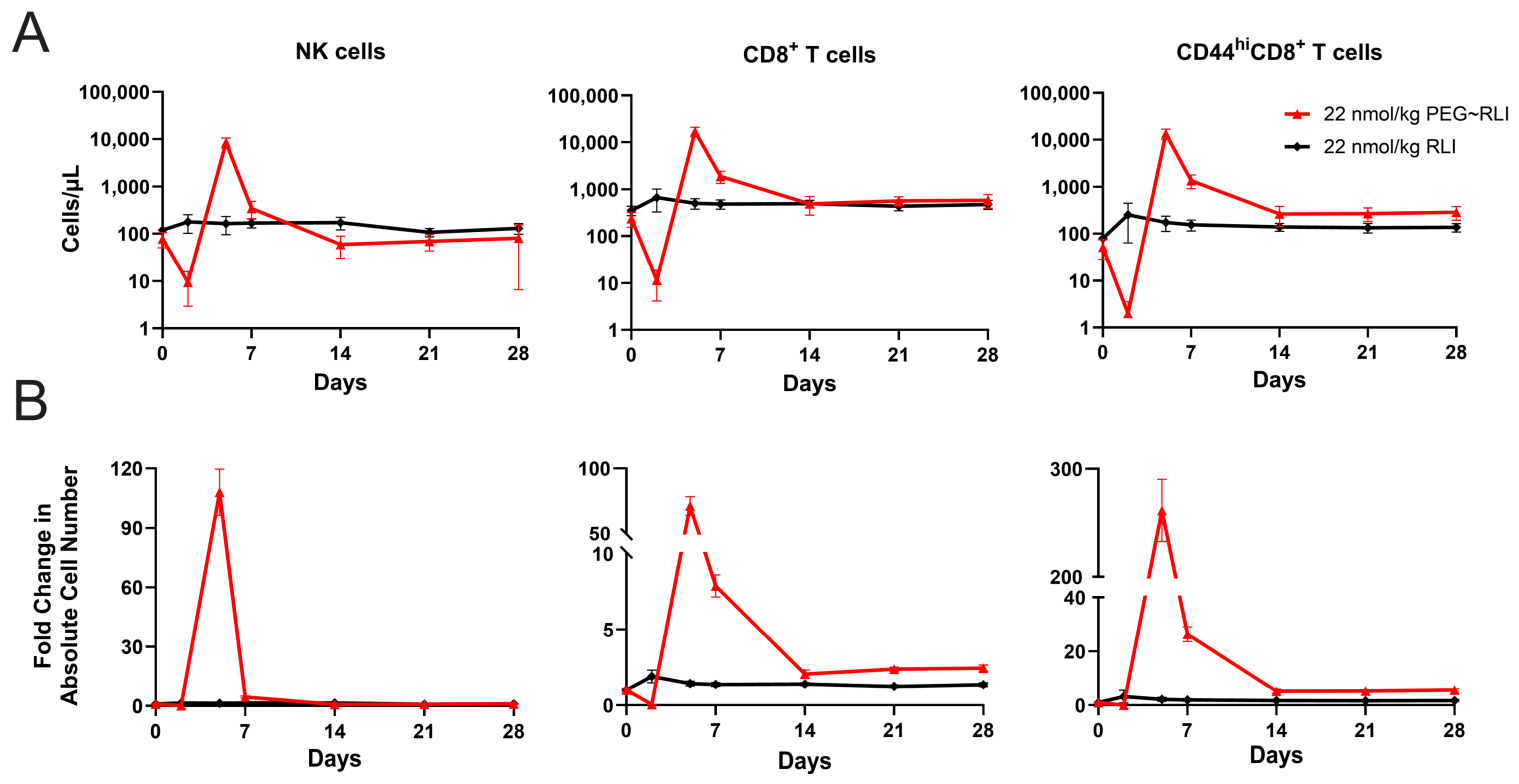
Longitudinal pharmacodynamic response of RLI and PEG-RLI. Time courses of NK, CD8^+^ T, and CD44^hi^CD8^+^ T cell expansion after treatment with 22 nmol/kg RLI (◆) or PEG-RLI (▲) (n = 5/group). PBMCs were sampled and immunophenotyped at −2, 2, 5, 7, 14, 21, and 28 days post-dosing. Panel (**A**) shows absolute cell counts; panel (**B**) shows the fold change in cell counts from the pre-bleed, derived from panel (**A**). Data points represent mean ± SD. Data are representative of three independent experiments.

PD effects of RLI, PEG-RLI and PEG-IL-15. We wanted to compare the PD response of PEG-RLI with other IL-15 agonists. We recognized that PD comparisons based on cell percentages can be misleading due to variability in baseline values and disproportionate effects of changes in cell populations. Thus, to facilitate comparisons with other experiments, we report both the fold-increase in absolute cell counts and the absolute number of cells at days 5 or 6. As previously described, 10 µg RLI (0.43 nmol/mouse; 22 nmol/kg)—equimolar to 6 µg IL-15—caused a modest 2-fold expansion in NK cells and CD8^+^ T cells and a 2.2-fold expansion CD44^hi^CD8^+^ T cells, similar to that obtained with ~25 µg IL-15 [8]. Animals treated with 4, 10, 20 and 40 µg PEG-RLI showed increasing expansion in immune cells up to 20 µg—with ~71% of maximal at 10 µg/mL and then a large decrease at 40 µg coincident with significant weight loss and lethargy (Figures S7 and S8). Single SC dosing of 10 µg PEG-RLI induced a robust systemic immune response over a 28-day period. In the longitudinal study, there was an initial drop in NK and CD8^+^ T cells, which we attribute to tissue migration [17,18,19,20], followed by a very high increase in absolute cell numbers (Figure 3A). At the response apex at day 5, there was a ~100-fold expansion in NK cells (8400/µL), ~78-fold for CD8^+^ T cells (16900/µL), and ~240-fold for CD44^hi^CD8^+^ T cells (13300/µL) (Figure 3B); a separate cross-sectional experiment at day 5 showed similar expansion of 63-fold of NK (4050/µL), 46-fold of CD8^+^(14000/µL), and 204-fold of CD44^hi^CD8^+^ T cells (11200/µL). In the prospective study, NK cell expansion persisted for ~7 days, while CD8^+^ and CD44^hi^CD8^+^ T cell expansions plateaued above control levels after day 14 and remained elevated. These expansions were all accompanied by 60 to 80% Ki67 expression (Figure S11). In spleen, there was a 10-fold higher level of NK cells and 12.5-fold more CD8^+^ and 11.6-fold more CD44^hi^CD8^+^ T cells than in PBMCs (Table S2). An experiment using 4 to 40 µg PEG-RLI prepared by the one-step synthesis (PEG_40kDa_-RLI) showed a similar pattern of immune cell expansion in peripheral blood on day 5 that peaked at 10 to 20 µg and dropped at the toxic 40 µg dose (Figure S9). The expansion was attenuated compared to that described above, and at 10 µg, it was about two-fold lower than PEG-RLI prepared by the two-step method; differences were attributed to different staining procedures and flow cytometers used.

**Figure 4:**
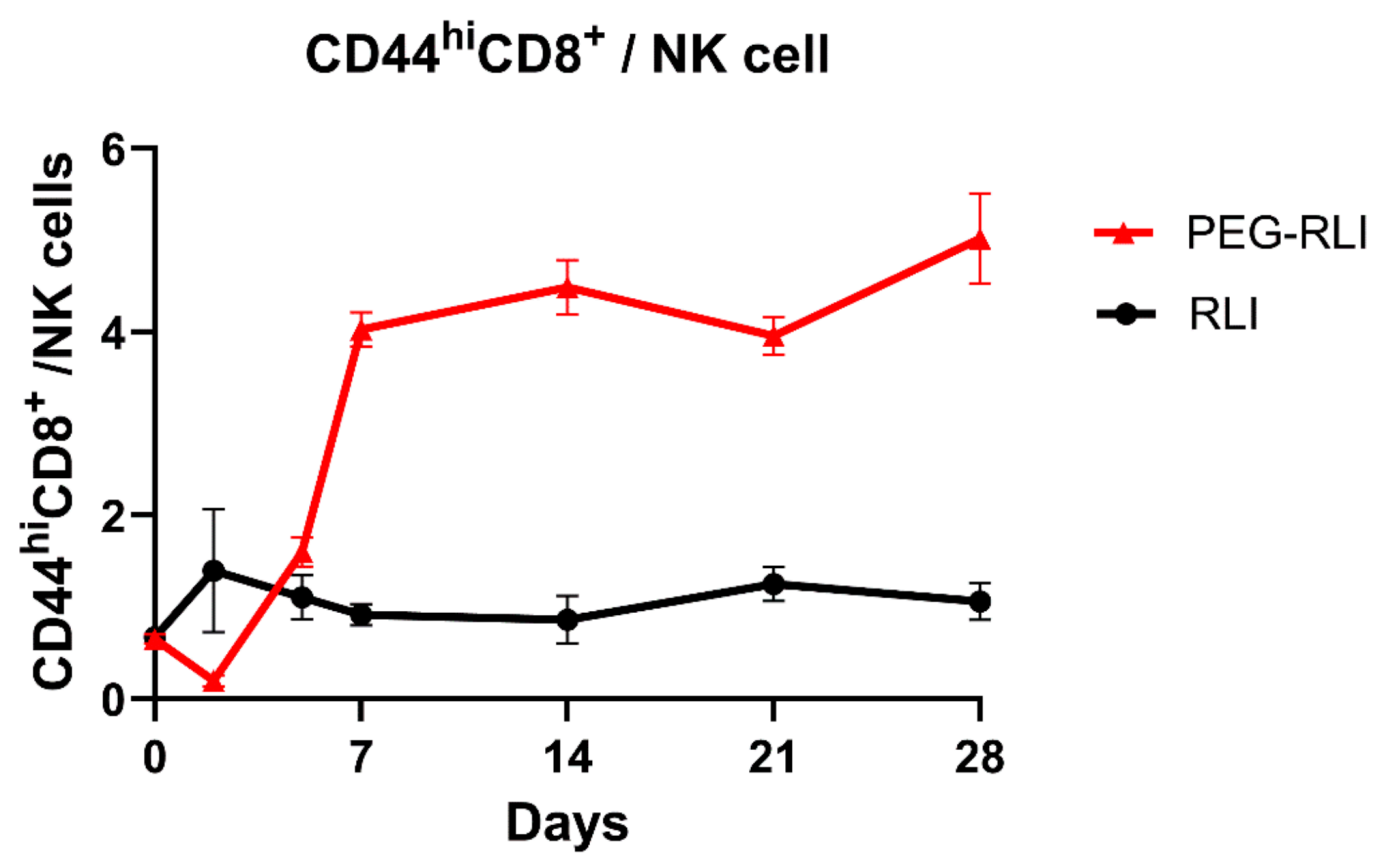
Ratios of CD44^hi^CD8^+^/NK cells in PBMCs. C57BL/6J mice were treated with either 22 nmol/kg PEG-RLI (▲) or 22 nmol/kg free RLI (⬤). All doses are expressed in terms of protein content. Data points represent mean ± SD.

With the exception of NKTR-255 [21] and reported MS depots [8,12], the data reported for other long-acting IL-15 agonists are insufficient to allow for quantitative comparisons to PEG-RLI. We could not obtain NKTR-255 for direct comparison to PEG-RLI because the relevant patent [22] did not identify which of several PEG-IL-15 conjugates prepared was NKTR-255. Nevertheless, we surmised that comparing fold expansions and ratios of immune cells over internal controls would provide consistent comparisons across experiments. NKTR-255 has been reported to cause significant expansion in immune cells [5]. At concentrations that cause maximal response, it induces a ~24- to 50-fold expansion in NK cells (~6000/µL), 6- to 8-fold expansion in CD8^+^ T cells (~1500/µL), and 20-fold expansion in CD44^hi^CD8^+^ T cells (~1000/µL) relative to vehicle control (Figure S13A,B). When fold expansion and immune cell ratios are compared, PEG-RLI showed ~2-, 11- and 13-fold greater expansion for NK, CD8^+^ T, and CD44^hi^CD8^+^ T cells, respectively, than that reported for NKTR-255. We also observed that at day 7 and after, the CD44^hi^CD8^+^/NK ratio was approximately 4-fold higher with PEG-RLI than with RLI or NKTR-255—consistent with relative enrichment in CD8^+^ T cells compared to NK cells (Figure 4 and Figure S14). Given differences in experimental conditions, comparison of PEG-RLI with NKTR-255 should be considered descriptive and hypothesis-generating rather than definitive.

Taken together, the results show that PEG-RLI induces a dose-dependent expansion in NK and CD8^+^ T cells, with ~200-fold increases observed for CD44^hi^CD8^+^ T cells at peak. Overall, PEG-RLI demonstrates much higher immune cell expansion metrics than its component RLI.

**Figure 5:**
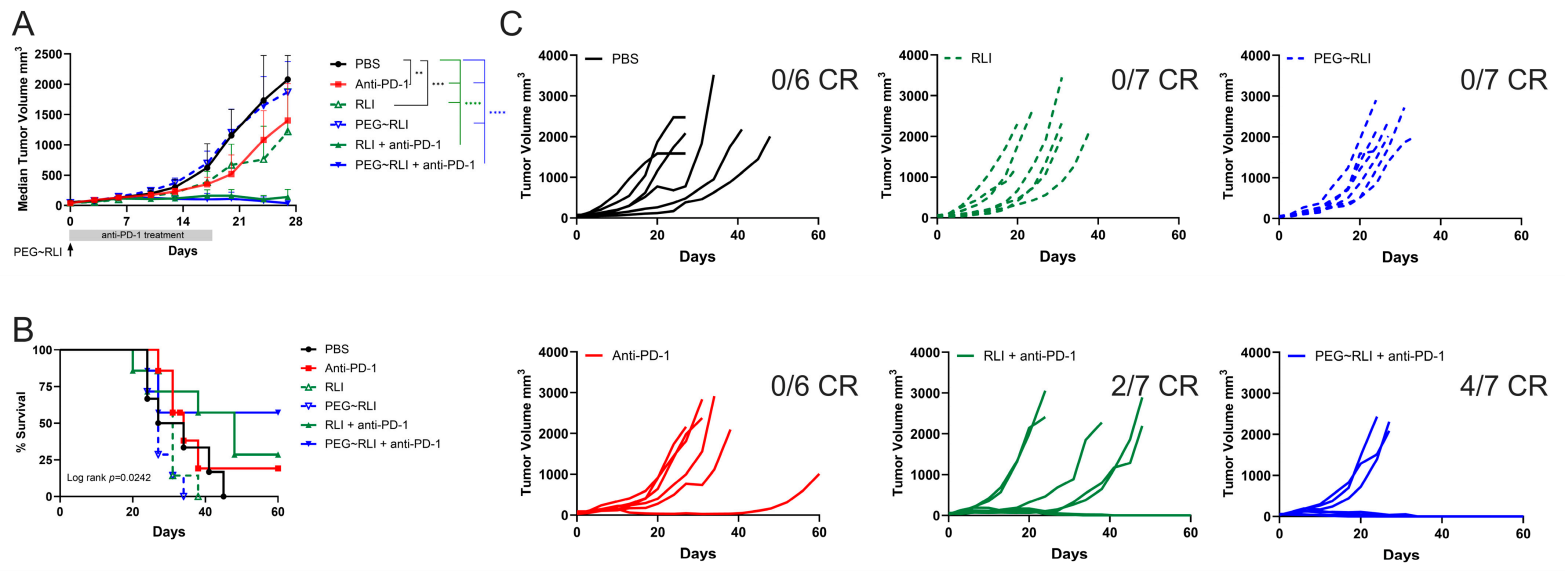
Efficacy of PEG-RLI and antiPD-1 in CT26 tumor-bearing mice. Mice bearing tumors with an average volume of 50 mm^3^ were administered PBS (⬤, n = 6), 0.5 mg/kg PEG-RLI SC (▽, n = 6), 0.5 mg/kg RLI SC (△, n = 6), and 200 µg anti-PD-1 IP (■, n = 6) on days 0, 5, 7, 13 and 17 or a combination of anti-PD-1 IP and RLI (▲, n = 7) or a combination of anti-PD-1 IP and PEG-RLI SC (▼, n = 7). (**A**) Tumor volumes are expressed as median ± median absolute deviation (MAD). Data were analyzed by two-way ANOVA, followed by Tukey’s post hoc test (** *p* ≤ 0.002; *** *p* ≤ 0.0002; **** *p* < 0.0001). (**B**) Kaplan–Meier survival analysis. Survival was monitored for up to 60 days. Overall survival differed significantly among treatment groups (log-rank [Mantel–Cox] test: χ^2^ = 12.92, df = 5, *p* = 0.0242). Pairwise log-rank comparison between PEG-RLI + anti-PD-1 and PEG-RLI yielded *p* = 0.04 (uncorrected). (**C**) Individual tumor volumes with complete responses (CR). Data are representative of a single experiment.

Efficacy of PEG-RLI plus anti-PD-1 in CT26 tumor-bearing mice. The antitumor efficacy of IL-15 agonists has been evaluated in a wide range of preclinical models [23], including CT26 murine colorectal carcinoma [24,25]. It was previously shown that RLI—but not IL-15—in combination with anti-PD-1 showed synergistic antitumor effects in the syngeneic CT26 colon carcinoma model [26]. We examined the antitumor efficacy of PEG-RLI in combination with anti-PD-1 in the same model. Following tumor establishment in the flank of female BALB/c mice, the animals were treated with single-agent RLI, PEG-RLI, anti-PD-1, or a combination of either RLI and anti-PD-1 or PEG-RLI and anti-PD-1. As analyzed by tumor growth, anti-PD-1 + RLI or anti-PD-1 + PEG-RLI caused moderate but unimpressive growth inhibition (Figure 5A). As analyzed by survival curves, a significant overall difference among treatment groups was observed (log-rank [Mantel–Cox] test: χ^2^ = 12.92, df = 5, *p* = 0.0242; n = 6–7 mice per group) (Figure 5B). The longest median survival was observed in mice treated with PEG-RLI + anti-PD-1 (51 days) compared with 27–31 days for the single-agent RLI, PEG-RLI and anti-PD-1. Pairwise log-rank comparison between PEG-RLI plus anti-PD-1 and PEG-RLI monotherapy yielded *p* = 0.04 (uncorrected), suggesting a trend toward improved survival with combination therapy. Together, these findings suggest a modest survival benefit associated with the combination regimen.

Given the variability in tumor growth kinetics among individual animals, analysis of responses in individual animals provided additional insight (Figure 5A,C). While no complete responses were observed in mice receiving single-agent therapy, four out of seven mice treated with PEG-RLI plus anti-PD-1 survived beyond 60 days and achieved complete tumor regression with no measurable tumor burden. Consistent with previous reports [11], the combination of RLI and anti-PD-1 also improved survival outcomes, although the effect was less pronounced, with two mice achieving complete responses.

Collectively, these data indicate that although single-agent PEG-RLI or anti-PD-1 confers limited efficacy in the CT26 model, their combination extends median survival and induces durable complete responses in a subset of treated animals. These results support the potential of PEG-RLI to enhance the therapeutic activity of immune checkpoint blockade with anti-PD-1.

## 4. Discussion

A number of long-acting IL-15 agonists are showing interesting activities in clinical trials: (1) NKTR-255, a PEGylated IL-15 [27]; (2) N-803 (Anktiva) and SHR-1501, IL-15 bound to an Fc-IL-15 Rα Sushi domain fusion [28,29]; (3) hetIL-15, native IL-15 and IL-15Rα [30] and (4) XmAb24306 (efbalropendekin alfa), an attenuated IL-15/IL-15Rα-Fc-Fusion [31]. This study characterized the new long-acting IL-15 agonist, PEG-RLI—a PEGylated IL-15/IL-15 Rα Sushi domain fusion—and compared its pharmacologic properties to those of other long-acting IL-15 agonists.

Because PEGylated IL-15, NKTR-255, has demonstrated encouraging clinical activity and RLI is substantially more potent than IL-15, we anticipated that a PEGylated RLI would retain high biological activity while gaining improved pharmacokinetics and pharmacodynamic properties. Structural analysis of the IL-15–IL-15Rα/βγ_c_ complex shows that the N-terminus of the Ra Sushi domain is distal to the IL-15Rβγ_c_ binding region of IL-15 [32]. We thus hypothesized that PEGylation of the N-terminus of RLI would have minimal impact on IL-15Rβγ_c_ binding. NKTR-255 was prepared by acylation of IL-15 with an activated PEG_40kDa_-ester [22], whereas our PEG_40kDa_~RLI was prepared by site-specific reductive alkylation of the N-terminus of RLI with MeOPEG-CHO. The PEGylated RLIs prepared here showed a modest 7-fold increase in EC_50_ from RLI in a cell-based receptor assay. PK evaluation in mice revealed significant improvements following PEGylation. PEG-RLI had an SC bioavailability of ~50% and a 15 h t_1/2_ in mice, 5-fold longer than the 3 h t_1/2_ of RLI and almost identical to the related PEG-IL-15, NKTR-255. The increased exposure (AUC) of PEG-RLI more than compensates for its modest reduction in receptor potency. Based on the PK profile, we calculate that the plasma concentration of PEG-RLI following a 10 μg dose would remain above an estimated therapeutic level of ~1 nM for 4 to 5 days, albeit with a C_max_/C_min_ ~250. Notably, the t_1/2_ of elimination of PEG_40kDa_ in humans is about five days, so the C_max_/C_min_ required to keep it above a 1 nM therapeutic threshold for five days would only be ~2. Since the projected C_max_/C_min_ needed to maintain PEG-RLI above human therapeutic levels is ~125-fold less than in mice, PEG-RLI should show significantly lower toxicity in humans than in mice.

PD assessments revealed similarities and differences of PEG-RLI compared to other IL-15 agonists. A single SC dose induced robust and sustained expansion of NK cells, CD8^+^ T cells and activated CD44^hi^CD8^+^ T cells in peripheral blood and lymphoid tissues. Consistent with its high potency, long t_1/2_ and high exposure, PEG-RLI produced substantially greater CD8^+^ and CD44^hi^CD8^+^ T cell expansion and exposure than free IL-15 or RLI. Despite inducing robust expansion in NK cells, CD8^+^ T cells, and CD44hiCD8^+^ T cells, PEG-RLI showed limited antitumor activity as a monotherapy in the CT26 model. These findings indicate that lymphocyte expansion alone is insufficient for tumor control. The enhanced antitumor effect observed with PEG-RLI plus anti-PD-1 highlights the importance of checkpoint blockade overcoming tumor-mediated suppression [24] and restoring or enhancing the antitumor functionality of the available effector lymphocytes expanded by PEG-RLI [26].

NK cells respond robustly to IL-15 agonists due to their high baseline expression of IL-15 receptor components. Indeed, IL-15 drives equal or greater NK cell expansion compared to CD8^+^ T cells, with NK cells showing up to ~50-fold higher sensitivity and more sustained proliferation [33]. Long-acting IL-15 agonists like NKTR-255 [21], XmAb24306 [34] and N-803 [25] also induce preferential or comparable expansion of NK cells compared to CD8^+^ T cells. While enhanced NK cell expansion may strengthen innate immune responses, excessive or sustained stimulation by IL-15 agonists has been associated with NK cell exhaustion and NK-cell-mediated toxicities [8,35,36,37].

While NK cell activation is valuable for early tumor control or hematologic malignancies, sustained adaptive immunity provided by CD8^+^ T cells is critical for scenarios requiring sustained antigen-specific responses and durable adaptive immunity [38]. However, the quality of the response—such as the cells’ functional ability (e.g., cytotoxic molecule production), spatial requirements (e.g., proximity to tumor cells), and resistance to suppression—also plays a crucial role. Provided that these CD8^+^ T cells retain cytotoxic function, effectively infiltrate tumors and resist immunosuppressive signaling [39], their increased systemic availability may enhance antitumor immune responses, as supported by prior studies [40,41,42]. Therefore, clinical scenarios that depend on robust adaptive immunity represent potential targets for agonists that stimulate expansion of CD8^+^ T cells. PEG-RLI preferentially expanded CD8^+^ and CD44^hi^CD8^+^ T cells, while only delivering moderate NK cell expansion, and may favor immunophenotypic conditions associated with adaptive antitumor immunity. Direct functional assessment of CD8^+^ T cell and NK cell activity, including cytotoxicity, activation status and tumor specificity, will be required to confirm this interpretation.

We posit that PEG-RLI produces distinctive responses compared to NKTR-255 and other IL-15 agonists because of its high potency and differential presentation to receptors. Although the C_max_, t_1/2_ and AUC of PEG-RLI and NKTR are nearly identical, the higher immune cell expansion by PEG-RLI is likely in part due to the higher affinity of RLI vs IL-15 for the receptor. Unlike IL-15, which requires endogenous cis or trans presentation [21], RLI covalently links IL-15 to the IL-15Rα domain complex and mimics trans presentation by directly engaging IL-15Rβγ_c_ receptors. This design stabilizes the complex, prolongs receptor occupancy and amplifies downstream STAT5-driven proliferation. The PK properties of PEG-RLI and PEG-IL-15, NKTR-255, are quite similar, yet PEG-RLI appears to be more effective at expanding immune cells. We posit that this is due to the continuous exposure of NK and CD8^+^ effector cells to the more potent agonist RLI compared to IL-15 and trans presentation’s therapeutic advantages of targeted activation.

CD8^+^ T cells serve as primary effectors in chronic viral infections, autoimmune diseases, transplant rejection, and cancer immunity, where they mediate cytotoxic responses against infected, self, or—most relevant here—malignant cells. Numerous preclinical models have provided critical insights into the mechanisms by which CD8^+^ T cells mediate antitumor immunity and may help refine the use of IL-15 agonists to enhance CD8^+^ T cell responses in various therapeutic settings. Preclinical studies have demonstrated synergy between IL-15 agonists and several immunotherapeutic modalities, including: (A) checkpoint inhibitors such as PD-1 and CTLA-4 antibodies, as shown in the TRAMP-C2 prostate cancer model [43]; (B) immune checkpoint stimulators exemplified by agonistic CD-40 in bladder cancer [44] and the TRAMP-C2 model [45,46]; (C) adoptive cell therapies, demonstrated with NKTR-255 in lymphoma-bearing mice [47]; and (D) stimulation of immune cell engagers like PD-1/IL-15 [48] and T cell engagers like the CD3 bispecific antibody anti-HER2/CD3 [34].

Some of these preclinical models have successfully translated into treatments for human cancers. Two exemplary clinical settings are highlighted from studies of the long-acting PEG-IL-15 agonist NKTR-255. First, IL-15 agonists are well known to demonstrate synergy with checkpoint inhibitors, overcoming immunosuppressive tumor microenvironments and enhancing tumor control. In the RESCUE trial, NKTR-255 combined with the PD-L1 inhibitor durvalumab significantly reversed radiation-induced lymphopenia in non-small-cell lung cancer patients following chemoradiation and restored absolute lymphocyte counts [16]. Second, in the context of adoptive cell therapies, NKTR-255-induced CD8^+^ T cells enhanced CAR-T cell persistence and efficacy, improving the durability of CAR-T therapy responses [27,49,50,51]. Administration of NKTR-255 after CAR-T therapy in patients with various hematological malignancies who had progressed following prior CAR-T cell treatment resulted in significant increases in response rates and CAR-T cell persistence. Although PEG-RLI has not yet been tested with CAR-T cells, its pharmacokinetic, structural and mechanistic similarities to NKTR-255 suggest that this combination warrants investigation.

Two additional clinical opportunities for PEG-RLI derive from studies of IL-15/IL-15Rα Sushi domain Fc fusion proteins, such as N-803, where the active moiety is shared with PEG-RLI and the Fc fragment is replaced by PEG for the intravesical treatment of non-muscle invasive bladder cancer (NMIBC) [43]. N-803 was recently approved as a combination therapy with BCG for the intravesical treatment of BCG-unresponsive NMIBC with carcinoma in situ (CIS). Here, N-803 enhances the proliferation and activation of NK cells and CD8^+^ T cells within the bladder tumor microenvironment and is synergized by BCG, leading to improved clinical outcomes and durable tumor remission. This combination activates both innate and adaptive immune responses—specifically dendritic cells and CD8^+^ T cells—within the bladder tumor microenvironment, leading to durable tumor regression. With PEG-RLI, the large hydrophilic PEG moiety should confer the two-hour maximum intravesical dwell time [52], and the RLI component is anticipated to stimulate CD8^+^ T cell responses as or more robustly than N-803, thereby providing effective antitumor responses.

Also, preclinical studies have shown that combining intravesical N-803 with an Fc-optimized anti-CD40 agonist antibody induces synergistic antitumor immunity in bladder cancer [44]. N-803 and CD40 agonists upregulate IL-15/IL-15Rα receptor complexes primarily on cross-presenting dendritic cells via CD40 stimulation, which then present IL-15/IL-15Rα to activate CD8^+^ T cells, leading to durable tumor-targeting immune responses. The large size of PEG-RLI may promote its retention in the bladder, and given its robust CD8^+^ T cell expansion, it is reasonable to anticipate that it may be quite effective when used in combination with agonistic anti-CD40 antibodies for intravesicular treatment of bladder cancer.

We posit that the high immune cell expansion observed with PEG-RLI in mouse models will translate to humans more faithfully than NKTR-255 and IL-15. In vivo studies of NKTR-255 and IL-15 in wild-type versus IL-15Rα-knockout mice demonstrated a pronounced 20–45% potency loss upon IL-15Rα ablation, underscoring the requirement for endogenous IL-15Rα for optimal activity [21]. STAT5 phosphorylation is a functional readout of IL-15 receptor engagement, where a strong STAT5 signal indicates effective receptor binding and activation of downstream signaling. The EC_50_ values for NKTR-255 and IL-15 in pSTAT5 induction increased approximately 8-fold in human versus mouse CD8^+^ T cells [5]. Thus, the reduced efficacy of IL-15 and NKTR-255 in mice compromises the murine model’s translational relevance for these agonists. In contrast, RLI—a fusion containing both IL-15 and IL-15Rα components—behaves as an IL-15Rα-independent cytokine that bypasses the requirement for endogenous IL-15Rα; it binds ~10-fold tighter to hIL-15Rβ than IL-15 [5] and showed strong STAT5 phosphorylation in both mouse and human cells, maintaining high-activity IL-15 trans-presentation even in xenogeneic contexts [26]. Hence, while NKTR-255 exhibits reduced activity in mice relative to humans, PEG-RLI’s murine activity is expected to robustly predict its human efficacy.

This study has several limitations. First, the efficacy and mechanistic findings were generated in preclinical models and will require further validation in clinical settings. Second, antitumor activity was evaluated in a single established tumor model, and broader assessment across additional tumor types and incorporating imaging-based approaches to assess tumor burden will help define the therapeutic potential of PEG-RLI as a single agent and in combination with anti-PD-1. In addition, our analyses focused primarily on pharmacodynamic and antitumor responses and did not include comprehensive characterization of safety [53], tolerability, intratumoral immune remodeling [54] or the activation and functional state of the expanded immune cell populations. Similarly, we did not exclude nonspecific immune effects of the PEG moiety itself; however, PEG alone is unlikely to directly induce CD8^+^ or NK cell proliferation or activation [55]. Future studies should include comprehensive safety assessment, including clinical chemistry, hematologic assessments, histopathology, characterization of systemic inflammatory responses, cytokine profiling, characterization of tumor-infiltrating immune populations and functional assessment of NK cells and CD8^+^ T cells, which will further define the therapeutic window and mechanisms of response. Finally, comparisons with other IL-15 agonists were based on published reports and should be considered exploratory. Direct head-to-head studies will be valuable for establishing relative pharmacologic and therapeutic properties.

In conclusion, we have prepared and characterized PEG-RLI, a long-acting IL-15 agonist that preferentially induces the expansion of CD8^+^ T cells. The number of CD8^+^ and CD44^hi^CD8^+^ T cells achieved with PEG-RLI is markedly higher than with other IL-15 agonists, including the related PEGylated IL-15, NKTR-255. These findings identify PEG-RLI as a promising IL-15-based immunotherapeutic, particularly in strategies requiring robust expansion of CD8^+^ T cells.

## Figures and Tables

**Table 1:** Distribution of PEG-RLI species following site-specific PEGylation.

	MeOPEG_20k_-CHO			MeOPEG_40k_-CHO			MeOPEG_2x20k_-CHO		
PEG Equivalents	1 eq	5 eq	10 eq	1 eq	5 eq	10 eq	1 eq	5 eq	10 eq
% Free RLI	54	17	19	86	64	43	86	51	51
% mono-PEG-RLI	43	67	63	13	33	51	13	44	44
% di-PEG-RLI	4	13	15	1	4	6	<1	5	4
% tri-PEG-RLI	0	2	2	0	0	<1	0	0	<1
% mono PEG-RLI of total modified	91	82	79	93	89	89	98	90	92

**Table 2:** Summary of pharmacokinetic parameters.

	Dosing Route	t_1/2_ h	C_max_, nM Normalized *	CL mL/h/kg	AUC_inf_, nM∗h Normalized *	F %	Reference
PEG-RLI	IV	15	11	2.8	360	100	This work
PEG-RLI	SC	15	4.4	2.8	160	46	This work
NKTR255	IV	15	34	2.3	370	100	[5]
MS~RLI	SC	30	0.009	NA	0.3	90	[12]
MS-IL-15	SC	170	0.001	NA	0.2	66	[8]
RLI	SC	3	0.1	145	7.5	NA	[15]
IL-15	IP	0.5	0.5	1600	0.8	NA	[15]
IL15	IV	0.17	34	510	2.0	100	[5]

* Values were dose-normalized to 1 nmol/kg.

## Data Availability

The original contributions presented in this study are included in the article/Supplementary Material. Further inquiries can be directed to the corresponding author.

## Supplementary Materials

The following supporting information can be downloaded at: https://www.mdpi.com/article/10.3390/pharmaceutics18070863/s1 [8,12,13,56,57], Figure S1. Reductive alkylation progress of RLI with azido-linker; Figure S2. Purification of PEG-RLI by anion exchange chromatography; Figure S3. PEGylation extent of RLI.; Figure S4. Purity of PEG40kDa -RLI; Figure S5. PEG-RLI bioactivity over 10 days stored at 37 °C, pH 7.4; Figure S6. PEG-RLI detection with IL-15 ELLA Simple Plex human IL-15 cartridge, of a PEG-RLI standard curve; Figure S7. Dose titration PD of PEG-RLI; Figure S8. Tolerability of PEG-RLI; Figure S9. Dose titration PD of PEG40kDa -RLI; Figure S10. Dose-dependent effects of PEG40kDa-RLI on body weight and cytokine profiles; Figure S11. Proliferation of target immune cells; Figure S12. Representative FACS plots showing the gating strategy used to analyze PBMCs from C57BL/6 mice that were left untreated (A) or treated with 10 µg PEG-RLI (B); Figure S13. Comparison of longitudinal pharmacodynamic responses of RLI, PEG-RLI and NKTR-255; Figure S14. Comparison of ratios of CD44hiCD8+/NK cells in PBMCs; Table S1. Antibodies used for immunophenotyping; Table S2. Spleen Weight and cell counts on day 5.
