# Supplementary material for: PEG-RLI: A Long-Acting IL-15 Agonist That Produces Massive Levels of CD8+ and CD44hiCD8+ Cells for Cancer Immunotherapy
Source: Pharmaceutics. 2026 Jul 15;18(7):863. doi: 10.3390/pharmaceutics18070863 (PMC13414841; doi:10.3390/pharmaceutics18070863)
Supplement: Supplementary file 1 [file pharmaceutics-18-00863-s001.zip › pharmaceutics-4385206-supplementary.pdf]

Supporting Information

PEG-RLI: A long-acting IL-15 agonist that produces massive levels of $CD8^+$ and $CD44^{hi}CD8^+$cells for cancer immunotherapy

Rocio del Valle Fernandez[1*], Guillermo Hails[1*], John A. Hangasky[1], Gary W. Ashley[1], Daniel V. Santi[1#]

[1] ProLynx Inc, San Francisco, CA, United States,
* Contributed equally

# Corresponding author: Daniel V. Santi, ProLynx, 135 Mississippi St., San Francisco, CA 94107; Daniel.V.Santi@prolynxinc.com. Phone 415 552 5306

Table of Contents

I. **General Materials and Methods**
   a. Materials
   b. Immunophenotyping
II. **Preparation and purification of PEG-RLI**
   a. Optimization of azido-linker-RLI yield
   b. Preparation of azido-linker-RLI
   c. Optimization of PEG-CHO reductive PEGylation of RLI
   d. Preparation PEG_{40kDa}-BCN
   e. Preparation of PEG-RLI
   f. Purification of PEG-RLI
   g. Preparation of PEG_{40kDa} -RLI at a large scale

III. **In vitro characterization of PEG-RLI**
   a. IL-2/IL-15Rβγ Receptor Dimerization
   b. Stability studies of PEG-RLI
IV. **In vivo studies**
   a. Animal welfare statement
   b. Preparation of dosing solutions
   c. Pharmacokinetic studies
   d. Pharmacodynamic studies
   e. Efficacy Study in CT26 tumor bearing mice
V. **Examples of flow cytometry plots for PBCMs immunophenotyping**
VI. **Supplemental References**

Supplemental Figures and Tables

Fig. S1. Reductive alkylation progress of RLI with azido-linker
Fig. S2. Purification of PEG-RLI by anion exchange chromatography
Fig. S3. PEGylation extent of RLI.
Fig. S4. Purity of PEG_{40kDa} -RLI.
Fig. S5. PEG-RLI bioactivity over 10 days stored at 37ºC, pH 7.4
Fig. S6. PEG-RLI detection with IL-15 ELLA Simple Plex human IL-15 cartridge, of a PEG-RLI standard curve
Fig. S7. Dose titration PD of PEG-RLI
Fig. S8. Tolerability of PEG-RLI

Fig. S9. Dose titration PD of PEG_{40kDa} -RLI
Fig. S10. Dose-dependent effects of PEG_{40kDa}-RLI on body weight and cytokine profiles.
Fig. S11. Proliferation of target immune cells
Fig. S12. Representative FACS plots showing the gating strategy used to analyze PBMCs from C57BL/6 mice that were left untreated (A) or treated with 10 µg PEG-RLI (B).
Fig. S13. Comparison of longitudinal pharmacodynamic responses of RLI, PEG-RLI and NKTR-255.
Figure S14. Comparison of ratios of $CD44^{hi}CD8^{+}$/NK cells in PBMCs.

Table S1. Antibodies used for immunophenotyping
Table S2. Spleen Weight and cell counts on day 5

I. General Materials and Methods

a. Materials. RLI was produced at ATUM (Newark, CA) based on previously reported methods (1, 2). antiPD-1, clone RMP1-14, was purchased from BioXcell. MeO-PEG_{20KDa}-CHO (Cat# ME-200AL), MeO-PEG_{40KDa}-CHO (Cat# ME-400AL2) and MeO-$PEG_{2x20KDa}$-CHO (Cat# BP2-400AL2) were purchased from NOF America; MeO-PEG_{40kDa}-NH_2 (Cat # 80506-64-5) was purchased from SINOPEG. All other chemicals and reagents were purchased from commercial vendors unless otherwise stated.

b. Immunophenotyping

Whole blood. EDTA whole blood (25 µL) was transferred to a 96 deep well plate and incubated with a fixable viability dye to label dead cells. FcRγII/II receptors were blocked with CD16/32 (20 µL, 1.6 µg) before staining for cell-surface antigens (all reagents from Invitrogen). The whole blood samples were incubated at 4°C for 30 minutes with previously determined optimal antibodies concentrations for surface staining of peripheral blood mononuclear cells (PBMCs) (**Table S1**). Red blood cells were lysed and PBMCs were fixed using 1-Step Fix/Lyse solution (Invitrogen). Fixed cells were washed once with permeabilization buffer and then intracellularly stained using a 30 minutes incubation period. After the cells were stained, samples were washed 2X with FACS buffer. The cells were resuspended in 400 µL FACS buffer and stored at 4°C until analysis.

Splenocytes. Single cell suspensions of splenocytes were obtained from harvested spleens following mechanical disruption and filtering through a 40-µm cell strainer. RBCs were then lysed using 1X RBC Lysis Buffer (Invitrogen) and ~$2.5x10^5$ splenocytes per sample were transferred to a 96 deep well plate. The FcRγII/III receptors were blocked with CD16/32 prior to staining for cell-surface antigens. Samples were incubated at 4°C for 30 minutes with previously determined optimal antibodies concentrations for surface staining of splenocytes (**Table S1**) and then, cells were fixed and permeabilized with the Foxp3 Transcription Factor Staining Buffer Set (Invitrogen) for intracellular marker staining following supplier instructions.

Stained single cell suspensions were read using a Attune NxT flow cytometer (BD Biosciences) and analyzed using FlowJo cytometry analysis software (TreeStar, Ashland, OR). The absolute cell numbers of the samples were determined by direct cell analysis (volumetric counting by the Attune NxT). The same gating strategy was followed as reported in Supplemental Ref. (3). Positive populations were identified based on fluorescence minus one control. CD8 and

CD44^{hi}CD8^{+} T cells were identified as CD3^{+}CD4^{-}CD8^{+} and CD3^{+}CD4^{-}CD8^{+}CD44^{hi}, respectively; NK cells were identified as CD3^{-}NK1.1^{+}. Cells with proliferative capacity were defined as Ki67^{+}.

Table S1. Antibodies used for immunophenotyping

Antibody	Clone	Vendor
Live/Dead Fixable Aqua dead cell stain	–	Invitrogen
CD3-FITC	145-2C11	Invitrogen
CD8a-PercP-Cy5.5	53-6.7	Invitrogen
CD4-eFluor780	GK1.5	Invitrogen
NK1.1-SuperBright600	PK136	Invitrogen
CD44-PE	IM7	Invitrogen
Ki67-APC	SolA15	Invitrogen
CD19-eFluor450	1D3	Invitrogen

II. Preparation and purification of PEG-RLI

a. Optimization of azido-linker-RLI yield

In a total volume of 75 µL, the reactions used 32.8 nmol (0.75 mg, 440 µM) RLI in 25 mM Citrate, pH 6.0, 500 mM NaCl and 0.05% tween-20 (Buffer Cit), 1 equivalent of N_3-PEG_4-CHO (16 µg, 440 µM) and 750 nmol (47 µg,10 mM) $NaCNBH_3$ and incubated at 4 ºC. Over a period of 24 hours, 5 µl samples were taken and diluted 20-fold in Buffer Cit + 50 mM β-Ala, to stop the reaction. Samples were treated with 0.5 mM DBCO-PEG_{5kDa} for 4 hours at 37 ºC and analyzed by the SDS-PAGE gel shift assay (4).

The reaction yields of time points along the reaction were assessed by densitometry. **Fig S1** shows the progression of the reaction. The mono-alkylated yield plateaus at six hours. Di-alkylated-RLI increased up to 3 % after 24 hours.

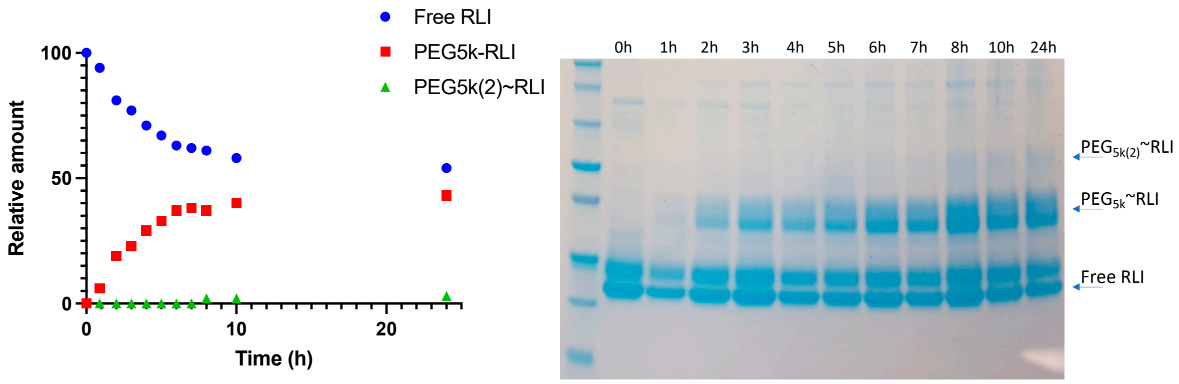

Figure S1. Reductive alkylation progress of RLI with N_3-PEG_4-CHO. Reaction timepoints were reacted with DBCO-PEG_{5kDa} and resulting species were analyzed by differential SDS PAGE gel

shift. Conversion to azido-linker-RLI plateaus at ~8 hours reductive alkylation time. Double alkylated RLI becomes detectable (~1%) after 8 hours of reaction at 4ºC.

b. Preparation of azido-linker-RLI

In 0.41 mL of Buffer Cit, reaction mixtures contained 217 nmol (5 mg, 530 µM) RLI, 217 nmol (75 µg, 530 µM, 1 Eq) of N_3-PEG_4-CHO and 5.2 µmol $NaCNBH_3$ (312 µg, 10 mM). The reaction was allowed to proceed 14 hours at 4 ºC in the dark. Excess reagents were removed using a 3.5 mL PD midiTrap G-25 column (Cytiva) previously equilibrated in 25 mM Tris-HCl, pH 8.0 (Buffer A). Protein concentration was determined by A280 (ε_{280nm}= 22960 $M^{-1}cm^{-1}$) using a NanoDrop spectrophotometer. SDS PAGE gel-shift analysis determined a mixture comprising 48% unreacted RLI + 40% mono-alkylated RLI + 12% di-alkylated RLI. Final buffer exchanged sample contained: 217 nmol of RLI (113 nmol alkylated RLI).

c. Optimization of PEG-CHO reductive PEGylation of RLI

In 30 µl reaction volume, 0.1 mg (4.6 nmol; 150 µM) RLI in Buffer Cit were mixed with 1; 5 or 10 molar equivalents (4.6, 23, 46 nmol) of either $MeOPEG_{40kDa}$-CHO, $MeOPEG_{20kDa}$-CHO, $MeOPEG_{2x20kDa}$-CHO or N_3-PEG_4-CHO and 300 nmol (19 µg,10 mM) $NaCNBH_3$. Reactions were incubated for 14 hours at 4 ºC and stopped by addition of 50 mM β-Ala. Control reactions containing N_3-PEG_4-CHO were further reacted with 0.5 mM $MeOPEG_{40kDa}$-BCN, for 4 hours at 37 ºC. Reductive alkylation yields were analyzed by SDS PAGE gel shift assay, loading reaction samples containing 3 µg of RLI/PEG-RLI species.

d. Preparation PEG_{40kDa}-BCN

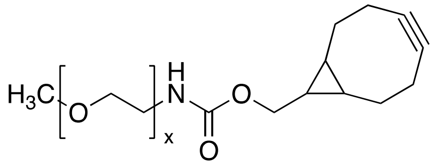

A mixture of MeO-PEG_{40kDa}-NH_2 (200 mg, 5 µmol), (1R,8S,9s)-bicyclo[6.1.0]non-4-yn-9-ylmethyl succinimidyl carbonate (BCN-OSu) (2 mg, 6.9 µmol, 1.4 Eq), and N,N-diisopropylethylamine (2 µL, 11.5 µmol, 2.3 Eq) in 2 mL of acetonitrile was kept for 30 min, then evaporated. The residue was dissolved in 2 mL of THF and precipitated by dropwise addition to stirred MTBE (10 mL). The precipitate was collected and dried under vacuum to provide the product.

e. Preparation of PEG-RLI

In 1.5 mL of 25 mM Tris-HCl pH 8.0, 170 nmol of MeO-PEG_{40kDa}-BCN (1.2 molar equivalents BCN:N_3) was added to the alkylated RLI reaction mixture of 217 nmol (~5 mg) of 40% mono-

alkylated RLI and 12% di-alkylated RLI. The reaction proceeded for 48 h at room temperature. The unreacted MeO-PEG-BCN was removed by incubation with 200 nmol of azido-Agarose beads (Click Chemistry Tools, Catalog # 1038-2) for 18 h at room temperature, followed by filtration using a 0.2 µm filters to remove both MeO-PEG_{40kDa}-Agarose beads and excess azido-Agarose beads.

f. Purification of PEG-RLI

HiTrap Q FF (7.5 x 0.7 cm, 3 mL column volume (CV), GE Healthcare) resin was equilibrated with 5 CV of 25 mM Tris-HCl, pH 8.0 (Buffer A). The final preparation mixture of PEG-RLI was loaded at 150 cm/h and unbound material was eluted with 3 CV of Buffer A. Using a flow rate of 150 cm/h, the sample was eluted using a linear gradient 0-10% 25 mM Tris-HCl, pH 8, 1 M NaCl (Buffer B) over 10 CV followed by a second linear gradient from 10% to 35 % Buffer B over 3 CV (**Fig S2**). Protein content and purity in collected fractions were analyzed by SDS-PAGE. Fractions containing purified PEG_{40kDa}-RLI were pooled, concentrated and buffer exchanged to PBS by diafiltration with a 10 kDa pore size Amicon filter. Purified and buffer exchanged PEG_{40kDa}-RLI was stored at -80 °C. SDS-PAGE was used to measure the purity (>95%) estimated by densitometry and free RLI was below the lower limit of detection (LLOD ~ 0.07 µg, or here <1.4 %). From the initial 5 mg of RLI material, 0.99 mg (24 %) were pooled as pure PEG-RLI and 1.88 mg (46 %) of unreacted RLI, implying 24 % process yield and 70 % overall recovery of starting material. By enabling the isolation and recycling of unreacted RLI, the well-resolved chromatographic step would permit iterative cycles of PEGylation and purification, thereby maximizing the overall reaction yield.

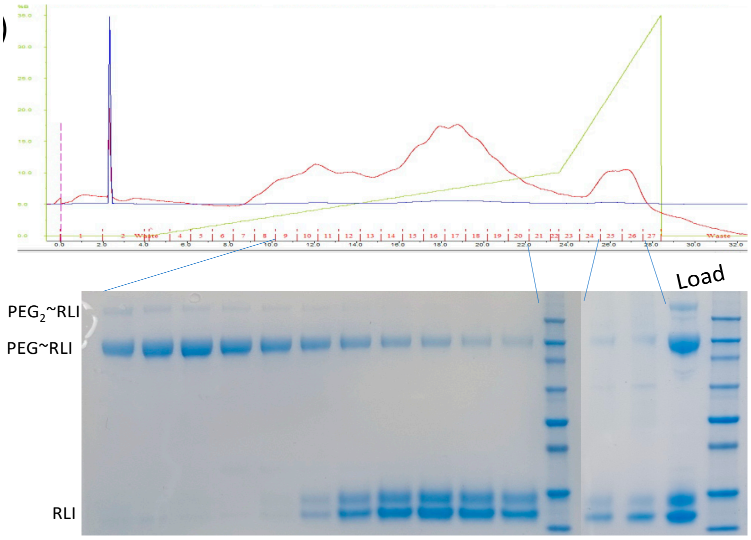

Figure S2. Purification of PEG-RLI by anion exchange chromatography. Chromatogram profile (A_{210nm}) was analyzed by SDS PAGE. Collected fractions 9- to 20 and 25- to 26 were compared to material loaded to the column. Fractions 9- to 14 containing PEGylated RLI and no detectable unmodified RLI were pooled. All lanes contained 5 µg total protein.

g. Preparation of PEG_{40kDa} -RLI at a large scale

In 4.0 mL of 25 mM Tris-HCl pH 8.1, RLI (41.1 mg, 1.8 µmol) was mixed with 5 eq. MeO-PEG_{40KDa}-CHO (360 mg, 9 µmol) and $NaCNBH_3$ (40 µmol, 10 mM). The reaction proceeded for 16 h at 0°C. The extent of PEGylation was analyzed by SDS PAGE gel shift assay, loading reaction samples containing 3 µg of RLI/PEG-RLI species. PEG_{40kDa}-RLI was purified by AEX, as described above. The purified and buffer exchanged PEG_{40kDa}-RLI was diluted to 0.43 mg/mL, aliquoted and stored at -80 ºC. From the initial 41 mg of RLI material, 6.23 mg (15 %) were pooled as pure PEG_{40kDa}-RLI. SDS-PAGE was used to measure the purity (>95%) estimated by densitometry and free RLI was below the lower limit of detection (LLOD ~ 0.07 µg, or here <1.4 %).

	% of Total
native	23%
monoPEG-RLI	58%
diPEG-RLI	16%
triPEG-RLI	2%

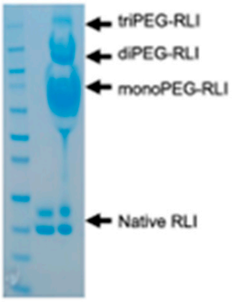

Figure S3. PEGylation extent of RLI. SDS-PAGE analysis of acylation of RLI with PEG_{40KDa}-CHO

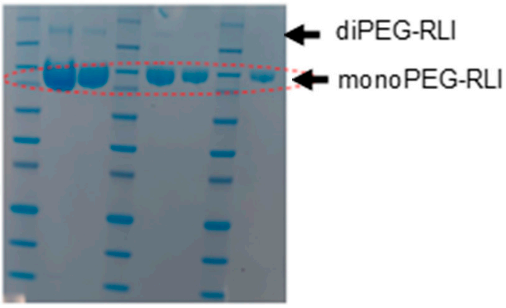

Figure S4. Purity of PEG_{40kDa}-RLI. Two-fold serial dilutions of PEG_{40kDa} -RLI (8 µg, 20 µL) were loaded to lanes 2, 3, 5, 6 & 8. Molecular weight marker was loaded to lanes 1, 4 and 7.

III. **In vitro characterization of PEG-RLI**

a. IL-2/IL-15Rβγ Receptor Dimerization.

A U2OS cell-based assay kit (DiscoverX, Part #93-0998E3CP5) for IL-2/IL-15Rβγ binding was performed according to the manufacturer's instructions and as previously described (5). U2OS

cells were plated (100 μL, ~5,000 cells/well) in 96 well assay plates and grown for 48 hours at 37ºC, 5% CO_2. Cells were then treated with PEG-RLI and incubated for an additional 6 hours at 37ºC, 5% CO_2. Ten PEG-RLI or RLI concentrations were assayed between 1.1 pM – 21.9 nM. Treated cells were incubated with a chemiluminescent substrate for 1 hour at room temperature protected from light prior to the luminescence being read with a Spectramax i3 plate reader using a 500 ms integration time.

b. Stability studies of PEG-RLI

The *in vitro* stability of PEG-RLI was assessed at pH 7.4, 37ºC for 10 days. PEG-RLI was incubated in PBS at 37 ºC and 10 μl aliquots were removed at 0, 1, 7 and 10 days and stored at -80ºC until the cell-based assay was performed.

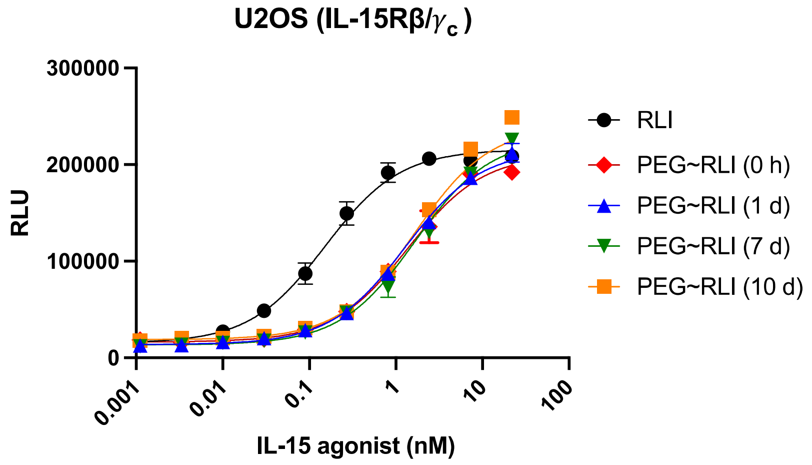

Figure S5. PEG-RLI bioactivity over 10 days stored at 37ºC, pH 7.4. The bioactivity of PEG-RLI was measured by its ability to induce IL-2Rβγ_c receptor dimerization. Aliquots were taken from a master reaction mixture at different timepoints: 0 hours [PEG~RLI (0 h);◆]; 1 day [PEG~RLI (1 d);▲], 7 days [PEG~RLI (7 d);▼] and 10 days [PEG~RLI (10 d); ■] and frozen at –80ºC until analysis; RLI (●) was used as a control. Data were fit to a three-parameter logistic model. Points represent the mean ± SD. The EC_{50} for PEG-RLI induced dimerization of IL-2Rβγ_c was determined to 1.4 nM for the 0 h time point and 1.7 nM for the 10-days' time point.

IV. In Vivo studies

a. Animal Welfare statement

This research complies with all relevant ethical regulations for animal testing and research. Animal handling and care was performed by MuriGenics (Vallejo, CA) and Explora Biolabs (San Francisco, CA). All animal studies were performed under protocols approved by the Institutional Animal Care and Use Committee of Murigenics or Explora Biolabs.

b. Preparation of Dosing Solutions

Dosing solutions were prepared by diluting the PEG-RLI stock in PBS. Concentration was confirmed by A_{280} (ε_{280nm}= 22960 $M^{-1}cm^{-1}$),

c. Pharmacokinetic studies

Doses containing 10 ug (0.43 nmol) of RLI were administered subcutaneously or intravenously to normal, male C57BL/J6 mice. Blood samples were collected in EDTA collection tubes, containing HALT protease inhibitor, over a predefined time course from alternating groups of mice (n = 3/ group). Plasma samples were prepared and stored at -80 ºC until analysis. The PEG-RLI concentration in plasma samples was assessed using the Simple Plex Human IL-15 Cartridge (Biotechne, Cat #SPCKB-PS-000500). A standard curve with PEG-RLI was performed as concentration reference. The PEG-RLI concentrations in plasma were plot as a function of time and fit using GraphPad Prism software.

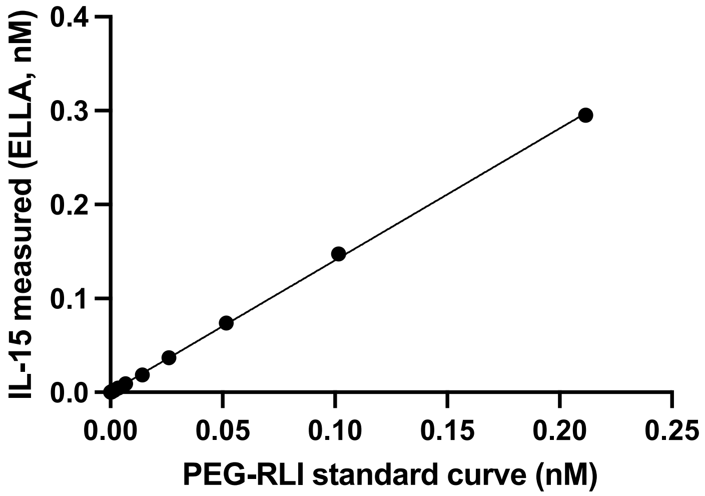

Figure S6. PEG-RLI detection with IL-15 ELLA Simple Plex human IL-15 cartridge, of a PEG-RLI standard curve. Measured IL-15 vs. PEG-RLI standard curve fits to a linear regression with a Y intercept of 0, slope of 1.405 and R^2 of 0.9996, indicating PEG-RLI can be accurately detected with this kit.

d. Pharmacodynamic studies

Dose titration pharmacodynamic of PEG-RLIs. Four groups of normal, male C57BL/6J mice consisting of 5 mice each with an average starting body weight of 25 g were injected SC with different quantities of PEG-RLI: 4 µg (0.17 nmol), 10 µg (0.43 nmol), 20 µg (0.86 nmol) or 40 µg (1.72 nmol). Body weights were determined twice a week. Blood samples were drawn on days -

2, and 5 and immunophenotyped using an Attune NxT Flow Cytometer. A multicolor panel was used to identify NK cells, $CD8^+$ T, and $CD44^{hi}CD8^+$ T cells as well as $Ki67^+$ subsets.

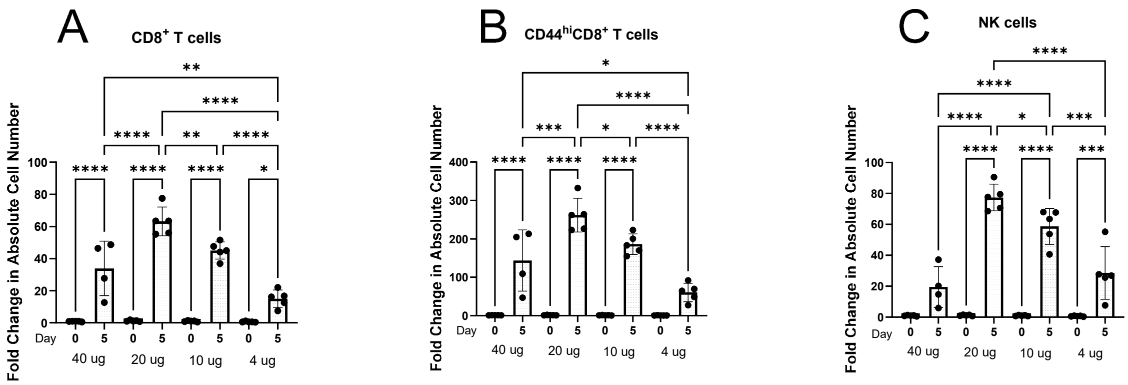

Figure S7. Dose titration PD of PEG-RLI. C57BL/6J mice (n = 4-5/group) were given a single SC injection of the conjugate at different doses: 4 µg (0.17 nmol), 10 µg (0.43 nmol), 20 µg (0.86 nmol) and 40 µg (1.72 nmol). At day 5 after treatment, a blood sample was collected and PBMCs immunophenotyped. The expansion of $CD8^+$ T cells (A), $CD44^{hi}CD8^+$ T cells (B) and NK cells (C) is expressed as fold change from the prebleed (d 0) samples**.** Data represents mean ± SD. Statistical analysis was performed using one-way ANOVA followed by Tukey´s post hoc test. *$p \leq 0.033$; **$p \leq 0.002$; ***$p \leq 0.0002$; **** $p < 0.0001$

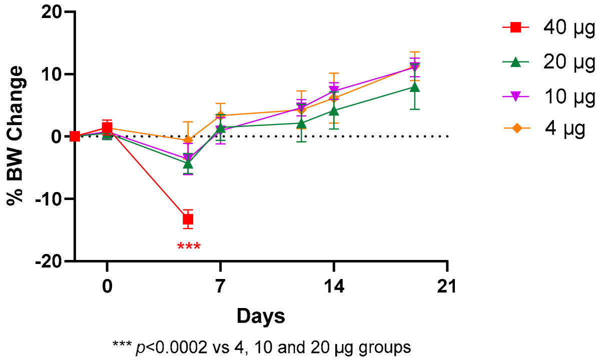

Figure S8. Tolerability of PEG-RLI. C57BL/6J mice (n = 5/group) were given a single SC injection of the conjugate and the body weights were followed over a 3-week period. Doses of 4 µg (0.17 nmol; ◆), 10 µg (0.43 nmol; ▼), and 20 µg (0.86 nmol; ▲) were well-tolerated. The highest dose of 40 µg (1.72 nmol; ■) showed ~15% body weight loss after 5 days. All dose weights are expressed in terms of protein content. Data represents mean ± SD. Statistical analysis was performed using mixed-effects analysis followed by Tukey´s post hoc test.

A similar study was conducted for PEG_{40kDa}-RLI (Figure S9). In this study six groups of normal, male C57BL/6J mice consisting of 6 mice each with an average starting body weight of 25 g were injected SC with the same quantities of PEG_{40kDa}-RLI as PEG-RLI. One group was treated with 10 µg (0.43 nmol) PEG-RLI as a direct comparison, and other group was left untreated. Body weights were determined twice a week. Blood samples were drawn on days 5 and 14, and immunophenotyped as previously described. In addition to immunophenotyping, plasma samples were collected, and an MSD customizable inflammatory panel (V-PLEX Mouse Proinflammatory Panel IFN-γ, IL-1β, IL-2, IL-6, IL-10, and TNF-α) was performed following provider's instructions.

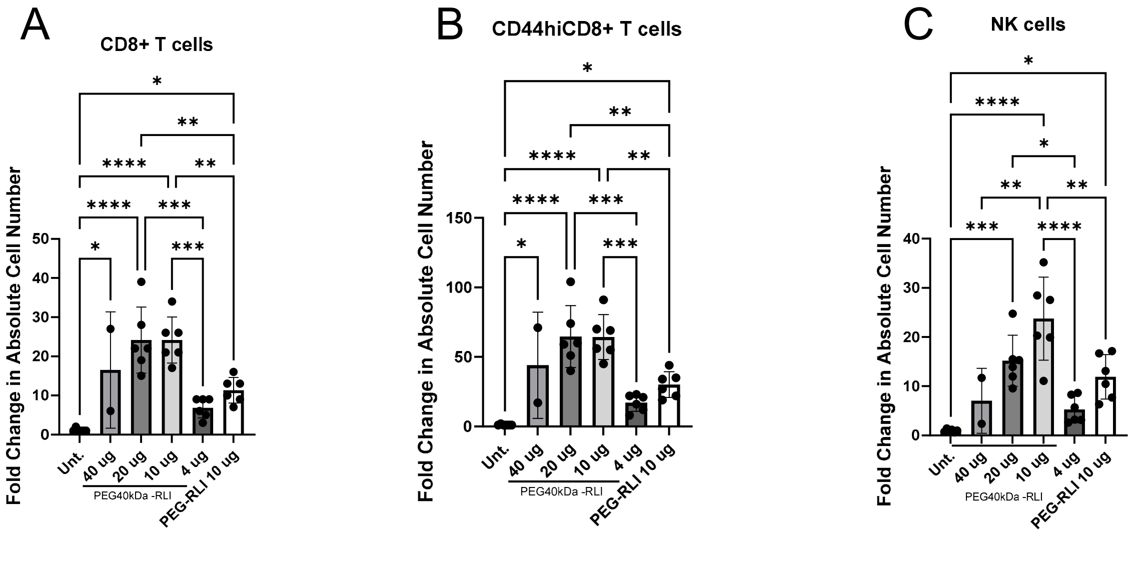

Figure S9. Dose titration PD of PEG_{40kDa}-RLI. C57BL/6J mice (n=6/group) were given a single SC injection of PEG_{40kDa}-RLI at different doses: 4 µg (0.17 nmol), 10 µg (0.43 nmol), 20 µg (0.86 nmol) and 40 µg (1.72 nmol) or left untreated. At day 5 after treatment, a blood sample was collected and PBMCs immunophenotyped. The expansion of $CD8^+$ T cells (A), $CD44^{hi}CD8^+$ T cells (B) and NK cells (C) is expressed as fold change from the Untreated (Unt.) group. Data represents mean ± SD. Statistical analysis was performed using one-way ANOVA followed by Tukey´s post hoc test. *$p \leq 0.033$; **$p \leq 0.002$; ***$p \leq 0.0002$; **** $p<0.0001$

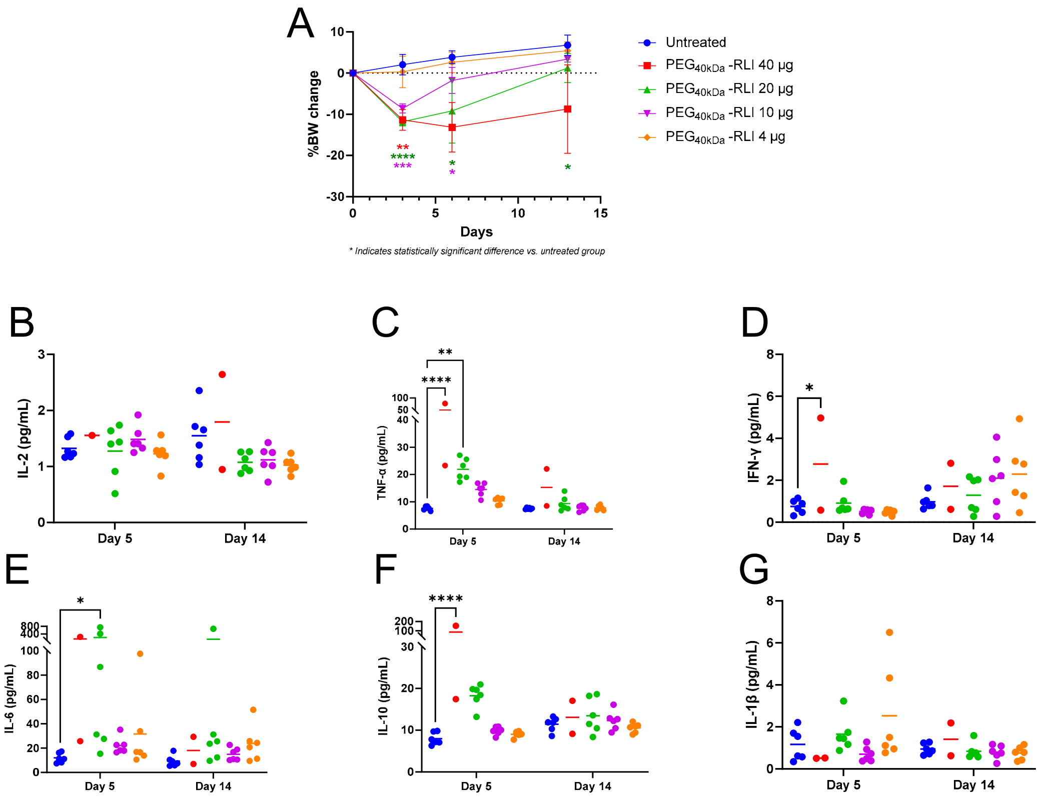

Figure S10. Dose-dependent effects of PEG_{40kDa}-RLI on body weight and cytokine profiles. C57BL/6J mice (n = 6/group) were given a single SC injection of PEG_{40kDa}-RLI at different doses: 4 µg (0.17 nmol), 10 µg (0.43 nmol), 20 µg (0.86 nmol) and 40 µg (1.72 nmol). A) Body weight expressed as mean percent body weight change over 13 days post-treatment. B-G) At days 5 and 14 after treatment, plasma samples were collected and the levels of IL-2, TNF- α, IFN-γ, IL-6, IL-1β and IL-10 were analyzed. Each point represents an individual mouse; bars indicate group means. Statistical analysis was performed using two-way ANOVA or mixed-effects analysis followed by Dunnett's post hoc test; significance is indicated vs. untreated group. *$p \leq 0.033$; **$p \leq 0.002$; ***$p \leq 0.0002$; **** $p < 0.0001$

Longitudinal pharmacodynamic response of PEG-RLI. A single dose of PEG-RLI or RLI (10 µg, 0.43 nmol) was administered SC to normal, male C57Black mice (n=5/group). On days -5, 2, 5, 7, 14, 21 and 28, were bled by submandibular blood collection and the PBMCs were immunophenotyped by flow cytometry.

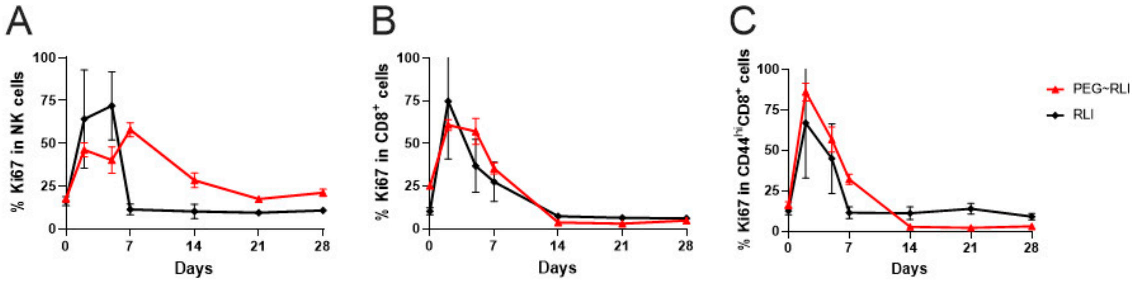

Figure S11. Proliferation of target immune cells. A) %Ki67$^+$ NK cells B) %Ki67$^+$ CD8$^+$ T cells and C) %Ki67$^+$ CD44^{hi}CD8$^+$ T cells following treatment with 22 nmol/kg RLI or PEG-RLI (n=5/group). Data points represent mean ± SD.

Pharmacodynamics of PEG-RLI in Spleen. Normal, male C57black mice (n=15/group) received either a single injection with 10 µg of PEG-RLI, or 10 µg of RLI SC On day 5, 7 and 14, 4 mice were sacrificed from each group while 3 were sacrificed on day 21. Spleens were harvested and single cell suspensions were prepared. Splenocytes were immunophenotyped to quantitate NK cells, CD8$^+$ and CD44^{hi}CD8$^+$ T as well as their proliferating subsets. Prior to the start of the study, five untreated mice were sacrificed to determine baseline cell numbers in spleen and blood.

Table S2. Spleen Weight and cell counts on day 5

	Untreated n=5	RLI (10 µg) n=4	PEG-RLI (10 µg) n=4
Average Spleen Weight (mg)	87.6 ± 6.4	76.5 ± 6.2	142 ± 19.4 $^{*;\#}$
NK cells / mg spleen	15400 ± 2200	15600 ± 3900	41000 ± 17900 $^{*;\#}$
CD8$^+$ T cells / mg spleen	189900 ± 28860	55400 ± 9800 *	174300 ± 39400 $^{\#}$
CD44^{hi}CD8$^+$ T / mg spleen	49800 ± 8760	16200 ± 3500 *	126900 ± 27700 $^{*;\#}$

Results are expressed as mean ± SD.
*Statistical analysis was performed using one-way ANOVA followed by Tukey´s post hoc test. *p≤0.05 vs untreated; # p≤0.05 vs RLI.*

e. *Efficacy study in CT26 tumor bearing mice*

CT26 tumors were established in the flank of female Balb/c mice via SC injection of CT26 tumor cells (1 x10^5 in 100 µL of serum-free medium). When tumor volumes reached ~50mm^3 (D0), mice treated with anti-PD-1 were administered one dose of anti-PD-1 (BioXcell, clone RMP1-14; 200µg, 20 µL); animals were excluded from the study if tumors responded to the initial anti-PD-1

treatment. On day 3, animals were randomized into groups (n=6-7/group): Group 1 was the control group (PBS). Group 2 was administered IP anti-PD-1 (200µg, 20 µL) on D0, D5, D7, D13, and D17. Group 3 and 4 were administered 0.5 mg/kg of free RLI or PEG-RLI SC, respectively, on day 4. Group 5 and 6 were given a combination of SC 0.5 mg/kg free RLI or PEG-RLI once on day 4 and IP anti-PD-1 D0, D5, D7, D13, and D17. Tumor volumes were measured (not blinded) two times a week until a humane endpoint was reached, and the overall survival of animals was recorded through 60 days.

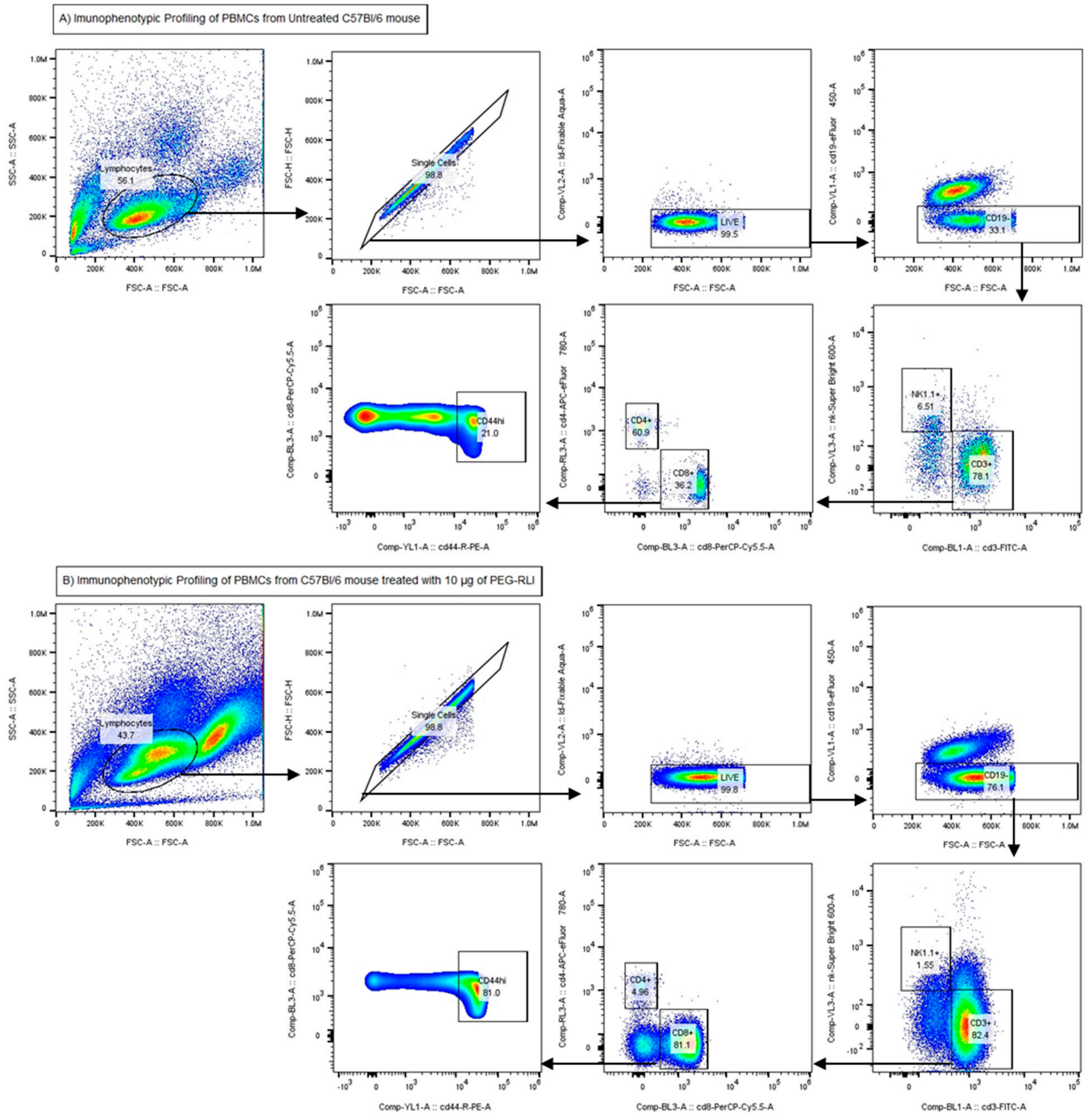

Figure S12. Representative FACS plots showing the gating strategy used to analyze PBMCs from C57BL/6 mice that were left untreated (A) or treated with 10 µg PEG-RLI (B). Lymphocytes were first gated based on FSC-A and SSC-A, followed by singlet discrimination using FSC-A vs FSC-H. Live cells were identified using a fixable viability dye. Live $CD19^{-}$ cells were then gated to exclude B cells. T cells were defined as $CD3^{+}$ cells and further subdivided into $CD4^{+}$ and $CD8^{+}$ subsets. CD44 expression was assessed within the $CD8^{+}$ compartment. NK cells were identified as $NK1.1^{+}$ cells within the $CD3^{-}$ population. Numbers shown in gates indicate the percentage of cells within the parent population. Plots are representative of the results presented in Figure S9.

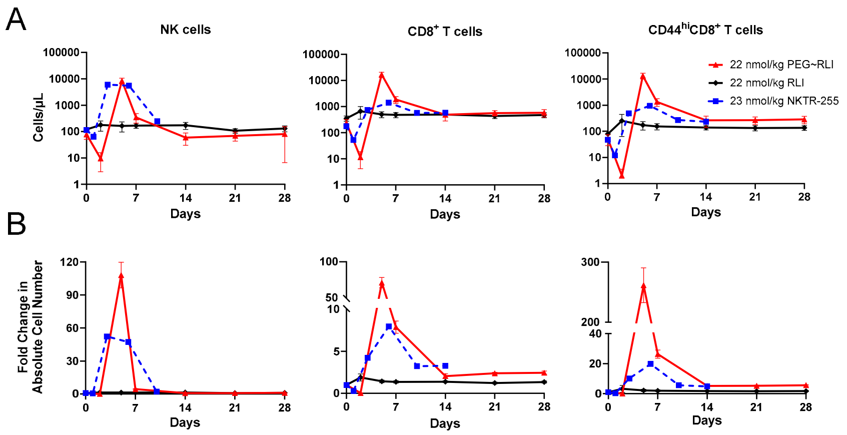

Figure S13. Comparison of longitudinal pharmacodynamic responses of RLI, PEG-RLI and NKTR-255. Time courses of NK, $CD8^{+}$ T, and $CD44^{hi}CD8^{+}$ T cell expansion after treatment with 22 nmol/kg RLI (♦) or PEG-RLI (▲) (n=5/group). PBMCs were sampled and immunophenotyped at -2, 2, 5, 7, 14, 21, and 28 days post-dosing. Data for NKTR-255 (■) was obtained by digitization of figures from Ref (5). Panel A shows absolute cell counts; Panel B shows the fold change in cell counts from the pre-bleed, derived from Panel A. Data points represent mean ± SD.

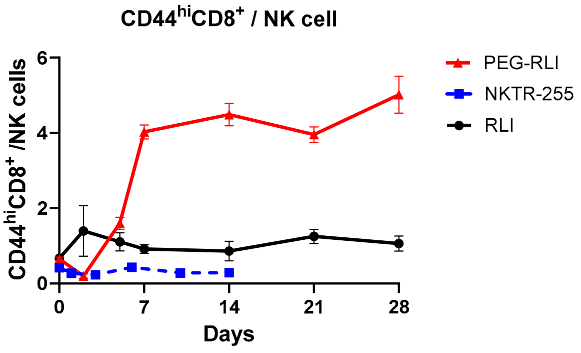

Figure S14. Comparison of ratios of CD44^{hi}CD8^{+}/NK cells in PBMCs. C57BL/6J mice were treated with either 22 nmol/kg PEG-RLI (▲), 22 nmol/kg free RLI (●). Absolute cell counts from Ref (5) were used to display NKTR-255 (■). All doses are expressed in terms of protein content. Data points represent mean ± SD.

V. Supplemental References

1. E. Mortier *et al.*, Soluble interleukin-15 receptor alpha (IL-15R alpha)-sushi as a selective and potent agonist of IL-15 action through IL-15R beta/gamma. Hyperagonist IL-15 x IL-15R alpha fusion proteins. *J Biol Chem* **281**, 1612-1619 (2006).
2. H. Perdreau *et al.*, Different dynamics of IL-15R activation following IL-15 cis- or trans-presentation. *Eur Cytokine Netw* **21**, 297-307 (2010).
3. J. A. Hangasky *et al.*, Leveraging long-acting IL-15 agonists for intratumoral delivery and enhanced antimetastatic activity. *Front Immunol* **15**, 1458145 (2024).
4. E. L. Schneider *et al.*, Approach for Half-Life Extension of Small Antibody Fragments That Does Not Affect Tissue Uptake. *Bioconjugate chemistry* **27**, 2534-2539 (2016).
5. J. A. Hangasky *et al.*, A very long-acting IL-15: implications for the immunotherapy of cancer. *J Immunother Cancer* **10** (2022).
